# A rare pre-existing progenitor-like Primed SMC compartment is the dominant inferred source of SMC-derived cellularity in vascular injury and atherosclerosis

**DOI:** 10.64898/2026.06.28.735042

**Published:** 2026-07-24

**Authors:** Shwethali Wani, Michael Kitching, Sobhan Aboulhassanzadeh, Teodora-Simona Lungu, Isil Kilicgun, Karsyn Ulibarri, Weimin Liu, Achilleas Floudas, Eileen M Redmond, Paul A Cahill

**Affiliations:** 1 Vascular Biology & Therapeutics, School of Biotechnology, Faculty of Science and Health, Dublin City University, Glasnevin Dublin, D09 K20V, Ireland; 2 Department of Surgery, University of Rochester Medical Center, 601 Elmwood Avenue, Box SURG, Rochester, NY 14642, USA; 3 Faculty of Medicine, School of Health Sciences, University of Ioannina, University Campus, 45110 Ioannina Greece

**Keywords:** Vascular smooth muscle cells, Atherosclerosis, Single-cell RNA sequencing, Lineage tracing, Clonal expansion, Phenotypic switching, CD34, Progenitor-like cell state

## Abstract

The cellular origin of smooth muscle cell (SMC)-derived populations in vascular lesions remains unresolved. Here we show, using single-cell transcriptomic analyses spanning carotid ligation injury, Myh11-CreERT^2^-traced aortic homeostasis, and LDLR- and ApoE-deficient atherosclerosis, that a rare progenitor-like "Primed" SMC compartment pre-exists at baseline in all models and in the healthy human aorta. Relative to contractile SMCs, Primed SMCs attenuate sarcomeric and contractile programmes while inducing matricellular, progenitor-niche and chondrogenic-poised developmental programmes, resolving into conserved niche/progenitor (*Cd34, Fst, Tnfrsf11b*) and matricellular (*Vcam1, Thbs1, Timp1*) cores overlaid by vessel-specific signatures, on a retained SMC identity. Multiple orthogonal computational lineage-inference approaches indicate that this compartment expands predominantly through autonomous self-renewal and is the dominant inferred source of cycling and lesion fibrochondrocyte populations, while contractile SMCs are consistently depleted as a feeder source. These findings reframe lesional SMC cellularity as expansion of a pre-existing Primed compartment rather than widespread phenotypic switching of contractile SMCs.

## Introduction

Atherosclerosis remains the principal cause of myocardial infarction and stroke worldwide, with smooth muscle cell (SMC)-derived cells constituting 40–70% of advanced lesion cellularity^1^. The prevailing paradigm holds that medial contractile SMCs undergo phenotypic switching, downregulating contractile markers and acquiring fibroblast- or macrophage-like identities to generate the diverse SMC-derived cells of lesions^2–4^. Myh11-CreERT^2^ lineage tracing showed that over 80% of SMC-derived lesion cells lack detectable contractile markers, and SMC-specific *Klf4* deletion reduces lesion size and thickens the fibrous cap, establishing KLF4-dependent modulation as central to pathogenesis^5,6^. Multicolour clonal tracing further established that SMC accumulation in atherosclerosis and carotid injury is oligoclonal, arising from extensive proliferation of a restricted subset of Myh11-expressing medial cells whose progeny generate the diverse lesion phenotypes^7–10^. This positions a small medial compartment, rather than the contractile majority, as the proximate source of plaque diversity, though its constitutive transcriptional identity and its relationship to candidate vessel-wall progenitors remain unresolved.

Single-cell analysis of healthy vessels identified a rare Sca1^+^/*Ly6a*^+^ SMC subpopulation (0.2–1.6% of medial cells) expressing disease-associated programmes^10,11^. Yet Sca1-CreERT^2^ tracing marks adventitial rather than medial SMCs and contributes minimally to lesional cells, with Sca1^+^/Pdgfra^+^ adventitial stem cells generating de novo SMCs only after severe injury^12,13^. Tracing of further candidate progenitors (*Gli1*^+^ adventitial cells^14–17^; *Sox10*^+^/*S100b*^+^ multipotent vascular stem cells^18–21^) has revealed context-dependent lesion contributions but has not resolved whether these populations overlap with constitutive *Myh11*-lineage SMC subpopulations. This creates a paradox in which scRNA-seq atlases consistently identify Sca1^+^ populations driving lesion expansion, yet Sca1-based tracing fails to label them. A statistical test of directional transcriptional ancestry against the reference pools available at each disease stage, replicated across independent models, has not been performed.

Here, using a murine carotid ligation cohort and three *Myh11*-lineage-traced datasets (healthy aortic media and longitudinal LDLR- and ApoE-deficient atherosclerosis), we identify a rare progenitor-like Primed SMC compartment present at homeostasis in every model and in the healthy human aorta. Relative to contractile SMCs, Primed SMCs attenuate sarcomeric and contractile programmes while inducing matricellular, niche/progenitor and chondrogenic-poised developmental programmes on a retained SMC identity. kNN feeder-source mapping — robust to cell-cycle structure and corroborated by pseudotime and rooted-tree trajectories — identifies a shared architecture across injury and both atherosclerosis models: autonomous expansion of the Primed compartment, depletion of contractile SMCs as a feeder source, and a Primed origin for the fibrochondrocyte and cycling populations. Because the compartment is recovered at baseline in all four cohorts, these analyses test continuity with a constitutive homeostatic compartment rather than retrospective similarity to a disease-emergent state. These findings reframe lesional SMC accumulation as autonomous expansion of a pre-existing Primed compartment rather than phenotypic switching of contractile SMCs, reconcile the Sca1^+^ paradox, and nominate intersectional *Myh11*-lineage tracing gated on *Cd34* or *Vcam1* as a decisive test of origin.

## Methods

### Animals and experimental models

All experimental procedures involving original mouse work for the carotid ligation cohort were approved by the University of Rochester Animal Care Committee and conformed to NIH guidelines for the care and use of laboratory animals.

### Carotid ligation model

Cdh5(PAC)-CreERT^2^ × Ai9 tdTomato reporter mice (Taconic Biosciences #13073 × Jackson Laboratory #007909) were used to generate Cdh5–Ai9 mice. Male mice aged 6–8 weeks (~20 g) were used; mice were housed under standard specific-pathogen-free conditions with a 12-hour light/dark cycle and food and water provided ad libitum. The Cdh5–Ai9 lineage-tracing background was part of the original experimental design but tdTomato transcripts were insufficiently detected in scRNA-seq data for reliable endothelial lineage assignment. The present study therefore defines cell identities transcriptionally.

Left common carotid artery ligation was performed as previously described^20,22^. Mice were anaesthetised with inhaled isoflurane, administered sustained-release buprenorphine (0.5–1.0 mg/kg, subcutaneous), and maintained on a temperature-controlled surgical platform. The left common carotid artery was exposed and ligated proximal to the bifurcation using 6–0 silk suture. Sham-operated mice underwent identical exposure without ligation. Arteries were harvested 14 days post-surgery for single-cell dissociation. For each condition, arteries from 10 mice were pooled and processed as a single 10x Genomics Chromium sample, yielding one pooled biological replicate per condition. This design provides averaged transcriptional information across 10 animals per group.

The sham and ligated arms analysed here form part of a wider multi-arm carotid study that additionally includes treatment arms reported separately. All arms were dissociated, sequenced, integrated and clustered together as a single object; the present study analyses only the untreated sham and ligated arms. The complete multi-arm object will be deposited on publication, with the treatment arms labelled Treatment X and Treatment Y.

Mice were euthanised by ketamine/xylazine anaesthesia followed by PBS perfusion. Common carotid arteries were dissected and immediately transferred into ice-cold FACS buffer (Thermo Fisher Scientific, Cat# 00–4222-57). Carotid artery segments from each sample were pooled and minced into fragments < 1 mm3 in enzyme digestion buffer containing Liberase TM (4 U/mL; Sigma-Aldrich, Cat# 540119001), hyaluronidase (60 U/mL; Sigma-Aldrich, Cat# H3506) and DNase I (120 U/mL; Thermo Fisher Scientific, Cat# 85836) in PBS, and incubated at 37 °C with shaking for 45–60 min. Samples were passed through a 70 μm strainer, centrifuged at 500 × g for 5 min at 4 °C, mechanically disrupted, rinsed with RPMI + 10% serum, re-pelleted and resuspended in RPMI + 10% serum. Viability was assessed by trypan blue exclusion. Dead cells were depleted using the Akadeum Microbubble Dead Cell Removal Kit. Final pellets were resuspended in RPMI + 10% serum prior to 10x Genomics library preparation.

### Healthy aortic medial SMC dataset

Healthy aortic medial SMC transcriptomes were obtained from GEO (GSE117963; GSM3316207)^10^. The present study analysed the four Myh11-CreERT^2^-marked (Confetti^+^) aortic medial 10x libraries (GSM3316207–GSM3316210; 12,092 lineage-positive cells). Because the Myh11-CreERT^2^ transgene is Y-linked, all mice were male. Tamoxifen was administered intraperitoneally (1 mg in corn oil) for 10 doses beginning at 6–8 weeks of age. Cells were enzymatically dissociated, and viable Confetti-positive singlets were isolated by FACS.

### Atherosclerosis lineage-tracing cohorts

Single-cell RNA-seq datasets from LDLR-deficient and ApoE-deficient atherosclerosis models were obtained from GEO (GSE155513)^23^. Myh11-CreERT^2^ mice (JAX 019079) were crossed with Ai6 (RCL-ZsGreen1) reporter mice (JAX 007906) and bred onto either Ldlr−/− (JAX 002207) or Apoe−/− (JAX 002052) backgrounds on C57BL/6J (JAX 000664). Tamoxifen-induced recombination permanently labelled SMCs and their progeny with ZsGreen1. Because the Myh11-CreERT^2^ transgene is Y-linked, all lineage-tracing mice were male. Mice received tamoxifen-containing chow for 2 days followed by 2 days of standard chow before initiation of a Western diet (Envigo Teklad TD.88137; 21% fat, 0.2% cholesterol) at 8 weeks of age. LDLR-deficient mice were analysed at 0, 8, 16 and 26 weeks of diet, and ApoE-deficient mice at 0, 8, 16 and 22 weeks.

The LDLR and ApoE cohorts were analysed as separate objects, each independently Harmony-integrated, embedded and clustered together with its own Western-diet timepoints, with no joint cross-cohort integration. The two cohorts share a single Control (pre-diet) timepoint rather than each carrying an independent Control: the same 6,562 Myh11-CreERT^2^ Control cells (3,506 FACS-negative and 3,056 ZsGreen^+^) were included in, and independently re-embedded and re-clustered within, each cohort object. Because every Control cell is common to both objects, the per-cohort Control Primed SMC counts (LDLR n = 141; ApoE n = 100) reflect independent clustering and lineage-gating of one shared baseline rather than two biological samples. The Western-diet timepoints are cohort-specific. Aortic tissue dissection was restricted to the ascending aorta, brachiocephalic artery and descending thoracic aorta. The aortic medial dataset sampled adventitia-free whole aortae^10^.

### scRNA-seq library preparation and alignment

#### Carotid ligation dataset

Pooled carotid arteries were processed using the 10x Genomics Chromium Single Cell 3′ v3 platform and sequenced on an Illumina platform at the University of Rochester Genomics Research Centre. BCL files were converted to FASTQ using Cell Ranger v7.0.1 mkfastq and aligned with Cell Ranger count to the mm10 reference (refdata-gex-mm10–2020-A) with the tdTomato reporter sequence appended (refdata-gex-mm10–2020-A-tdTomato). Cell Ranger recovered 2,285 sham and 4,925 ligated cells; median per-cell metrics were 4,569 genes and 18,643 UMIs (sham) and 3,378 genes and 10,989 UMIs (ligated). After quality control and doublet removal, 1,973 sham and 4,272 ligated cells were retained for downstream analysis (6,245 cells analysed; sham and ligated arms of the 26,426-cell multi-arm object).

#### Public datasets

Raw FASTQ files from public datasets were downloaded from the SRA and processed using a unified pseudoalignment workflow with kallisto v0.51.1, bustools v0.44.1 and kb-python v0.29.1 (10x v2/v3 workflow^24,25^). All datasets were aligned to the 10x Genomics mm10 2020-A reference. For the atherosclerosis cohorts, the ZsGreen1-WPRE reporter sequence was appended to the reference to enable simultaneous quantification of endogenous transcripts and lineage reporter expression. Filtered count matrices were generated in h5ad format and imported into Seurat v5.5.0 using zellkonverter^26^. Ensembl identifiers were converted to mouse gene symbols using org.Mm.eg.db. for downstream variable-feature analyses. The human aortic atlas was derived from the original Cell Ranger alignment^27^. Although Cell Ranger was used for the in-house carotid and human aortic datasets and kallisto/bustools for public datasets, both approaches yield comparable gene-level quantification.

Independent acute-injury dataset and marker-capture analysis. An independent 3-day carotid-ligation dataset (sham and ligated; SRA PRJNA628067)^28^ was downloaded and processed through the same pseudoalignment, normalisation, Harmony integration and clustering pipeline described above; the Primed and contractile SMC compartments were identified by the conserved Primed module (*Vcam1, Timp1, Thbs1, Cd34*) and the contractile module (*Myh11, Myocd, Cnn1, Carm*n), respectively. Marker-capture analysis was then applied uniformly across all five murine datasets (carotid ligation, aortic media, LDLR−/−, ApoE−/− and the 3-day ligation cohort). Within the baseline (sham/control) Primed and contractile compartments — restricted, for the lineage-traced atherosclerosis cohorts, to ZsGreen1-WPRE–positive cells — the fraction of cells expressing each candidate marker (*Cd34, Vcam1, S100b*) was computed as the proportion with detected transcript (counts > 0), an intentionally conservative lower bound on promoter activity given single-cell dropout. Percentages are reported with Wilson 95% confidence intervals, and Primed-versus-contractile enrichment as the ratio of these fractions. Reported single-cell percentages from the smooth-muscle–derived transient progenitor cell study^29^ were taken from that study's published single-cell re-clustering of the SMC compartment; those data were not reprocessed here.

#### Quality control and filtering thresholds

Quality control was performed independently for each cohort, with parameters chosen according to the cell-loss biology of each preparation. For all four cohorts, cells were filtered using nFeature_RNA and nCount_RNA outlier thresholds defined per sample as the 75th percentile + 1.5 × IQR, requiring a minimum of 200 detected genes per cell and a minimum of 3 cells per gene. Doublets were identified and removed using DoubletFinder (v2.0.6)^30^ with an expected doublet rate of 5%, pN = 0.25 and pK selected by parameter sweep maximising the mean-variance-normalised bimodality coefficient. Mitochondrial transcript fraction was quantified against the mouse mitochondrial gene set and applied uniformly as an exclusion criterion across all four cohorts at 10%. Cells exceeding 10% mitochondrial content were removed as likely dissociation-damaged. Mitochondrial transcript fractions were generally low across the carotid atlas after QC, with most clusters concentrated below the 10% exclusion threshold. Elevated mitochondrial content was restricted to a small number of discrete clusters rather than broadly affecting the major contractile SMC compartments, supporting the interpretation that Primed SMC recovery and expansion were not driven by high-mitochondrial damaged-cell artefacts. As a robustness check, applying a stricter ≥450-gene threshold removed none of the contractile or Primed SMCs analysed, all of which were high-complexity cells (per-cohort median 1,357–3,634 genes), leaving the Primed compartment, its module separation and *Cd34/Vcam1* enrichment unchanged.

#### SMC lineage assignment

In the healthy aortic medial dataset, all analysed cells had been pre-enriched for *Myh11* lineage identity by FACS in the original study and were treated as lineage-positive without further filtering; in healthy aorta, cell death and phagocytic clearance are minimal, so reporter enrichment is not appreciably confounded by protein transfer. For the LDLR-deficient and ApoE-deficient cohorts, FACS-sorted ZsGreen1-positive and ZsGreen1-negative fractions were merged prior to analysis and lineage was assigned by reporter transcript rather than by the sort gate. FACS reports ZsGreen protein, which in atherosclerotic plaques can be acquired both through Cre-mediated recombination and through phagocytic engulfment of ZsGreen1-positive cells and debris. Notably, cells discordant for the sort gate and the transcript (FACS-positive, transcript-negative) were predominantly myeloid, expressing macrophage markers (*Cd68, Adgre1, Lyz2, Csf1r, C1qa*) with the *Myh11*-positive contractile signature absent, consistent with phagocytic acquisition of reporter protein rather than heritable labelling (Supplementary Fig. 1). Defining lineage by the integrated transgene transcript excludes these false-positives and recovers genuinely recombined cells mis-assigned to the negative sort gate.

Lineage-positive cells were defined by ZsGreen1-WPRE expression exceeding 3.17 log-normalised counts. This threshold was set by maximising Youden's J against the FACS sort gate in the LDLR reference cohort (n = 26,673 cells; AUC = 0.90, sensitivity = 0.76, specificity = 0.97) and applied globally to both atherosclerosis cohorts and across all timepoints (Supplementary Fig. 2). FACS-positive and FACS-negative cells were clustered jointly (Harmony integration over sample) to provide a common transcriptional reference, and lineage status was assigned within that shared embedding. Because this criterion requires detectable transgene transcript, it is deliberately conservative.

#### Normalisation, integration and clustering

Count matrices were normalised using Seurat NormalizeData (scale factor = 10,000). Highly variable genes were identified with FindVariableFeatures (3,000 genes; VST), followed by ScaleData, and PCA was computed on the 3,000 variable features (50 PCs). Within each dataset, libraries (and timepoints, for the atherosclerosis cohorts) were integrated using Harmony v1.2.03^31^. Harmony used sample and timepoint metadata for the atherosclerosis cohorts and sample alone for the carotid and aortic medial datasets. UMAP embeddings and nearest-neighbour graphs were generated from Harmony-derived dimensions 1–41 (carotid), 1–45 (aortic medial), and 1–30 (LDLR and ApoE). Clustering used FindClusters at resolution 0.4 for all murine cohorts, chosen to resolve the principal mural, adventitial, endothelial and immune compartments, including the contractile SMC and Primed SMC compartments at single-population granularity, without fragmenting into sub-clusters lacking distinct marker profiles. At the clustering resolution used (0.4), fibromyocytes and fibrochondrocytes formed a single FbC cluster in the LDLR dataset and two adjacent clusters in the ApoE dataset; for cross-model comparability the two ApoE clusters were treated as a single FbC compartment in all composition, feeder and trajectory analyses. Each dataset was analysed independently; no joint cross-dataset Harmony integration or co-clustering was performed. Human aortic data were processed in two stages. All vessel-wall cells were normalised, scaled, and integrated across donors with Harmony, with UMAP embedding and clustering on Harmony dimensions 1–30 (resolution 0.5). Vascular SMCs were then subset and independently re-embedded and re-clustered across donors on Harmony dimensions 1–20 (resolution 0.3).

#### Differential expression and module scoring

Differential expression was performed using Seurat FindMarkers with Wilcoxon rank-sum testing, min.pct = 0.1 sets the prevalence floor for a gene to be tested, not a threshold on reported detection and logfc.threshold = 0.25 (for cross-cohort tier and enrichment analyses FindMarkers was run unfiltered and the log2FC > 0.25 cutoff applied post hoc, giving the same significant-gene set). Within-cohort tests used Bonferroni correction (default Seurat output); cross-cohort comparison families used Benjamini–Hochberg correction across the four-cohort family. Genes with adjusted p < 0.05 and log2FC > 0.25 were considered significant. Module scores were calculated with AddModuleScore. The Primed SMC module comprised *Vcam1, Timp1, Thbs1* and *Cd34;* the strict contractile SMC module comprised *Myh11, Cnn1, Myocd* and *Carmn*; and the fibrochondrocyte (FbC) module comprised *Acan, Sox9, Col2a1, Comp, Chad and Ibsp*. Figure-specific extended panels — a seven-gene contractile panel (adding *Tagln, Acta2* and *Lmod1*) used for aortic-cohort module scoring and the contractile-SMC subclustering, and an expanded Primed panel for the aortic visualisations — were applied where indicated. Scores were compared using BH-adjusted pairwise Wilcoxon tests.

Cross-cohort signature architecture: Conserved Primed SMC signatures were identified by intersecting significantly up-regulated genes across all four cohorts (BH-adjusted p < 0.05, log2FC > 0.25, expressed in ≥ 10% of Primed SMC cells per cohort). Genes meeting cross-cohort criteria were stratified into pan-vascular conserved cores versus bed-specific axes by cohort-restricted enrichment patterns.

Cross-cohort Primed SMC versus contractile SMC differential expression was performed on n = 101 (sham carotid), n = 240 (aortic medial), n = 141 (LDLR Control) and n = 100 (ApoE Control) Primed SMC; lineage-positive cells were defined by ZsGreen1-WPRE > 3.17. Because the LDLR and ApoE cohorts share a single Control timepoint, the LDLR-Control and ApoE-Control comparisons are the same baseline cells re-clustered within each cohort object rather than independent samples, so the four comparisons span three independent biological baselines. Significance was assessed by two-sided Wilcoxon testing with Benjamini–Hochberg correction across the comparison family.

Cross-cohort cluster similarity. ClusterFoldSimilarity (CFS v1.80)^32^ was applied to the QC-filtered, independently log-normalised cohort objects, restricted to baseline/uninjured cells (sham carotid; control LDLR/ApoE; all healthy aortic medial), lineage-gated where applicable (ZsGreen>3.17 for LDLR/ApoE; Confetti-sorted medial cells for the aorta), and to the shared mesenchymal cell-type roster using final cohort-specific cluster annotations. Although clusters were defined on Harmony-integrated embeddings, similarity scores were computed solely from shared-gene fold-change signatures of the uncorrected RNA assay (13,830 common features; topNFeatures = 5, nSubsampling = 30) and did not use Harmony embeddings or batch-corrected expression. Compositions were compared at native depth and after downsampling to 120 cells per cell type (minimum 12).

#### Functional enrichment analyses

Differentially expressed genes from each Primed SMC versus contractile SMC comparison were partitioned into upregulated (BH-adjusted p < 0.05, log2FC > 0.25) and downregulated (BH-adjusted p < 0.05, log2FC < −0.25) lists. Over-representation analysis (ORA) was performed independently on each list using clusterProfiler v4.16.0^33^: enrichGO (OrgDb = org.Mm.eg.db v3.22.0^34^) for Biological Process and Cellular Component ontologies, and enrichKEGG (organism = “mmu”). Mouse gene symbols were converted to Entrez identifiers with AnnotationDbi::select against org.Mm.eg.db; unmapped symbols (Ensembl IDs, RIKEN cDNAs, predicted genes) were excluded, and the mapped DE-tested gene set served as the per-cohort background universe. Significance thresholds were pvalueCutoff = 0.05 and qvalueCutoff = 0.2 with Benjamini–Hochberg correction^35^. Terms reaching BH-adjusted p < 0.05 in at least one cohort were retained for cross-cohort visualisation; near-redundant terms with substantially overlapping gene sets were de-duplicated to the canonical term. In the cross-cohort dot plots, dot size encodes gene ratio and dot fill encodes signed −log10(BH-adjusted p), the sign reflecting enrichment in the upregulated (positive) or downregulated (negative) list.

All four cohorts (carotid, aortic medial, LDLR and ApoE) were analysed by ORA. The cross-cohort GO Cellular Component and KEGG dot-plots include all four cohorts. The GO Biological Process dot-plot is restricted to the three cohorts with a disease arm (carotid, LDLR and ApoE).

#### Cell-origin and trajectory analyses

kNN feeder-source analyses were performed in 20-dimensional Harmony space (a lower-dimensional space than clustering, to stabilise the neighbour-distance metric); UMAP coordinates were used only for visualisation.

#### kNN feeder-source statistical framework

To test whether disease-associated populations arise predominantly from autonomous expansion of pre-existing subpopulations rather than de novo phenotypic switching, a unified kNN feeder-source framework was applied to the carotid ligation, LDLR-deficient and ApoE-deficient datasets. For each query cell, the k = 15 nearest neighbours were identified among earlier-condition reference cells (sham for the carotid cohort; preceding timepoints for the atherosclerosis cohorts) in 20-dimensional Harmony space, and source-cluster contributions (“feeder fractions”) were compared with expected reference-pool proportions as an observed/expected enrichment. Bootstrap confidence intervals were computed from 50 bootstrap iterations and enrichment p-values from 500 permutations of the reference-cluster labels (one-sided, Laplace-corrected), with Bonferroni correction across reference clusters within each query. Reference sets were chosen per question: SMC-lineage queries (Primed SMC, Cycling Primed SMC, AdvFb1) used a mesenchymal-restricted reference (contractile SMC, Primed SMC, AdvFb1, AdvFb2, pericyte, mural), whereas the Mac negative control used the full reference. Multi-k sweeps (k = 5–50) were performed as sensitivity analyses. Small query sets (e.g. Cycling Primed SMC, n ≈ 20) are reported with direction and confidence intervals and treated as suggestive; sets of fewer than five cells were not tested. This framework tests transcriptional ancestry as the closest pre-injury or pre-disease state to each query cell, rather than clonal lineage.

Cycling Primed SMC were defined as ligated Primed SMC that were both in S/G2M phase (Seurat CellCycleScoring with the cc.genes.updated.2019 sets, title-cased for mouse) and positive for a mitotic module (*Mki67, Top2a, Birc5* > 0); this phase-and-module intersection (~20 cells) avoids the over-calling of phase alone.

#### Trajectory inference

Trajectory topology was inferred with Slingshot using dual rooting as a falsification test^36^. Each lineage was computed with the root placed alternately at the contractile/SMC cluster and at the Primed SMC cluster. Slingshot establishes connectivity and topology only; lineage directionality is adjudicated by the kNN feeder analysis, not by the trajectory, and pseudotime is presented as a topological ordering rather than a direct lineage trace. Lineage inference (getLineages) and pseudotime (getCurves) were computed in Harmony space — 20 dimensions on the carotid mesenchymal compartment, and 10 dimensions on the LDLR and ApoE SMC-fate compartments (SMC/Primed SMC/FbC/Cycling); curves were embedded to UMAP for display. For the carotid cohort, a cell-cycle-regressed re-integration (regressing S and G2M scores, re-running Harmony) confirmed that the topology is invariant to cell-cycle structure.

##### Independent reclustering validation

To validate the Primed SMC identity independently of the integrated sham–ligated atlas, sham carotid cells passing QC (1,973 cells) were reclustered separately in a sham-only Harmony embedding. Seven clusters were identified at resolution 0.4 and assessed for Primed SMC marker expression using AddModuleScore; reciprocal barcode mapping between the sham-only clustering and the integrated-atlas annotation confirmed the Primed SMC identity independently of the joint embedding.

Mitochondrial DNA (MtDNA) variant analysis. Mitochondrial genotypes were called from the carotid ligation library using the alignment generated at sequencing and processed with mgatk. Per-cell, per-position allele counts were imported with ReadMGATK (Signac v1.17.1; Seurat v5.5.0) cells with mean mtDNA coverage ≥ 5 were retained (2,227 cells), of which 1,300 mapped to annotated ligated populations (SMC, Primed SMC, Cycling Primed SMC, Cycling Myeloid, AdvFb). Candidate substitutions were enumerated with IdentifyVariants across the mouse mitochondrial genome (all 48,897 possible substitutions; 3 alternate alleles × 16,299 bp) and filtered under a high-confidence setting (detected in ≥ 5 cells, strand correlation ≥ 0.65, VMR > 0.01; the standard Signac criteria) and a relaxed exploratory setting (≥ 3 cells, strand correlation ≥ 0.50, VMR > 0.005). No variant passed either filter: a single site was confidently detected in ≥ 3 cells and it fell below the dispersion threshold (VMR ≤ 0.005), so no site satisfied both the detection and dispersion criteria and the strand-correlation requirement never became the deciding factor. Mean alternative-allele frequencies sat at the noise floor (≈ 10^−4^) despite adequate per-position coverage (up to ~150×), with strand correlation undefined for nearly all sites as expected for 3′ GEX data. No heteroplasmy-based clonal reconstruction or population-enrichment testing (positivity threshold 10% heteroplasmy) was therefore performed.

To assess whether the inferred SMC-fate architecture was specific to the kNN-feeder or Slingshot methods, the SMC-fate clusters (contractile, Primed, and where present fibromyocyte, fibrochondrocyte and cycling) were re-embedded independently (PCA followed by Harmony integration on sample) and analysed by two orthogonal approaches. For partition-based graph abstraction, a connectivity value was computed for each cluster pair as the ratio of observed to expected edges in the k-nearest-neighbour graph, with expected edges derived from a degree-preserving null model that controls for cluster size^37^. For temporal-flow analysis, the k = 30 nearest earlier-stage neighbours of every later-stage cell were identified in the shared embedding for each adjacent timepoint pair, and the cluster identity of those neighbours tabulated; enrichment was expressed as observed versus expected neighbour composition given the earlier-stage cluster frequencies. This cross-timepoint neighbour analysis is conceptually related to optimal-transport ancestor–descendant inference^38^.

### Statistical analysis

Analyses were performed in R v4.6.0 on macOS Apple Silicon (aarch64-apple-darwin23), with Seurat v5.4.0. Exact adjusted p-values are reported; values below the double-precision limit are reported as p < 2.2 × 10^−308^. Permutation feeder p-values are Laplace-corrected and floored at the permutation resolution ((1/501) × n_ref).

Within-cohort differential-expression p-values were Bonferroni corrected; cross-cohort DE and functional-enrichment p-values were Benjamini–Hochberg corrected. Wilcoxon rank-sum tests were used for non-parametric cell-level comparisons because single-cell expression distributions are zero-inflated and non-normal. Feeder bootstrap analyses used 50 iterations and permutation tests 500 iterations, with Laplace-corrected empirical p-values. Cluster proportions are reported as percentages of total cells (carotid) or lineage-positive cells (atherosclerosis cohorts). The carotid cohort was designed as a discovery cohort with arteries pooled from 10 mice per condition, providing averaged transcriptional information across animals; compositional changes are therefore reported descriptively without formal replicate-based statistical modelling. Biological-replicate-level statistical power for the Primed SMC compartment was provided by three independent replication cohorts: Dobnikar et al. 2018^10^ (12,092 marked-only cells analysed, from a 24,440-cell dataset across 8 libraries); Pan et al. 2020 LDLR (26,673 cells total; 11,956 lineage-positive cells called at the >3.17 threshold, 11,362 retained in the atlas after restriction to the mural/SMC-lineage compartments and QC); and Pan et al. 2020 ApoE (24,042 cells total; 10,988 lineage-positive cells called at the same threshold, 9,015 retained after the same restriction and QC)^23^. In both atherosclerosis cohorts, cells were pooled per timepoint with no biological replicates – the only libraries are the two FACS sort fractions (ZsGreen^+^ and ZsGreen^−^) so per-timepoint compositional changes are reported descriptively rather than tested across replicates. Convergent recovery of the Primed SMC signature and the autonomous-expansion architecture across these independent cohorts indicates that the findings are not artefacts of the pooled carotid design. No statistical methods were used to predetermine sample size. Group allocation for surgery (sham vs ligated) was not randomised and surgical procedures were not blinded, owing to the clear anatomical distinction; downstream computational analyses were performed without prior knowledge of subsequent comparisons but were not formally blinded.

### Data availability

Original carotid ligation scRNA-seq data have been deposited in GEO under accession [GSE to be assigned]. The complete multi-arm carotid object (26,426 cells) is deposited with the treatment arms labelled Treatment X and Treatment Y; the underlying treatment-arm identities are released on publication of the intervention study. Public datasets reanalysed here are available from GEO accessions GSE117963 (healthy medial SMCs; GSM3316207) and GSE155513 (LDLR-deficient and ApoE-deficient cohorts). The independent 3-day carotid-ligation dataset is available from the SRA under accession PRJNA628067.

### Code availability

Custom analysis scripts implementing the kNN feeder-source framework, the Slingshot trajectory analyses and the marker-capture analysis have been deposited in a Zenodo repository under an MIT licence. The DOI [to be provided upon submission].

## Results

### A rare Primed SMC compartment is present in the healthy carotid artery

**Fig. 1.**
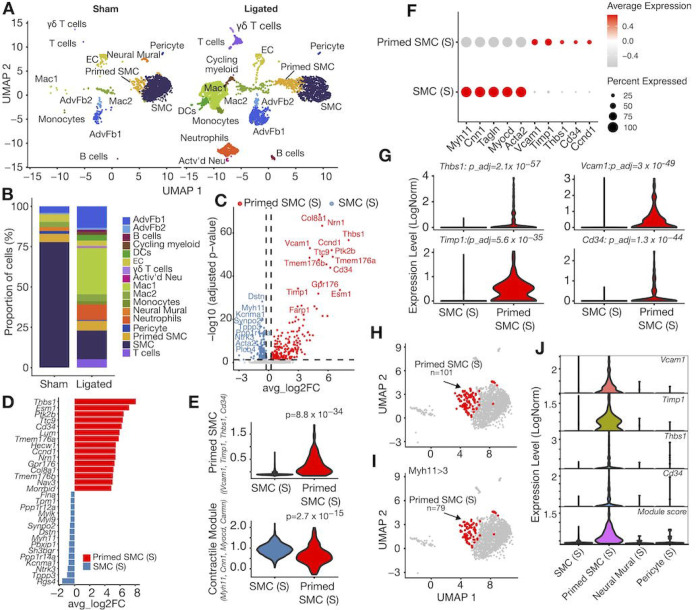
A rare Primed SMC compartment is present in the healthy carotid artery and expands after ligation. (A) Harmony-integrated UMAP of scRNA-seq from murine carotid arteries 14 d after ligation (Sham, n = 1,973; Ligated, n = 4,272 cells; 10 mice per group, pooled per capture), showing 17 clusters annotated by canonical markers (Supplementary Table 1). (B) Cell-type composition in Sham versus Ligated arteries: contractile SMC contract, while Mac1/Mac2 macrophages, AdvFb1 fibroblasts and Primed SMC expand and disease-associated populations (Cycling myeloid, DCs, neutrophils, activated neutrophils) emerge. (C) Volcano plot, sham Primed SMC versus sham contractile SMC (Wilcoxon two-sided, BH-corrected): red, enriched in Primed SMC; blue, enriched in SMC; grey, n.s.; selected genes labelled. (D) Top differentially expressed genes ranked by avg log_2_FC. (E) Primed SMC module score (*Vcam1, Timp1, Thbs1, Cd34*; upper, p = 8.8 × 10^−34^) and contractile module score (*Myh11, Cnn1, Myocd, Carmn*; lower, p = 2.7 × 10^−15^) in sham SMC versus sham Primed SMC (Wilcoxon, BH-corrected). Scores are background-corrected relative values (AddModuleScore); negative values do not denote absent expression. (F) Dot plot of module-gene expression in sham SMC versus Primed SMC (dot size, % expressing; colour, mean expression). (G) Per-gene violins of the four module genes (*Thbs1* p_adj = 2.1 × 10^−57^; *Vcam1* 3 × 10^−49^; *Timp*1 5.6 × 10^−35^; *Cd34* 1.3 × 10^−44^; Wilcoxon, BH-corrected). (H) UMAP of the 101 sham Primed SMC. (I) The 79 sham Primed SMC passing the *Myh11* > 3 log-normalised gate (red; 78.2%) on the full sham UMAP (grey); the gate is a transcriptional SMC-identity proxy. (J) Module-gene score (*Vcam1, Timp1, Thbs1* and *Cd34)* violins in the Myh11-gated population, showing Primed SMC-specific enrichment relative to SMC, Neural Mural and Pericyte.

Single cell RNA sequencing (scRNA-seq) analysis of murine carotid arteries 14 days after sham operation or ligation (n = 10 mice per group, pooled per capture) yielded 1,973 sham and 4,272 ligated cells after QC, resolving 17 Harmony-defined clusters (Fig. 1A,B; Supplementary Table 1). Sham arteries were dominated by quiescent contractile SMCs (77.7%) but also harboured a smaller, transcriptionally distinct SMC population present at homeostasis, hereafter Primed SMC (n = 101; 5.1%).

**Extended Data Fig. 1.**
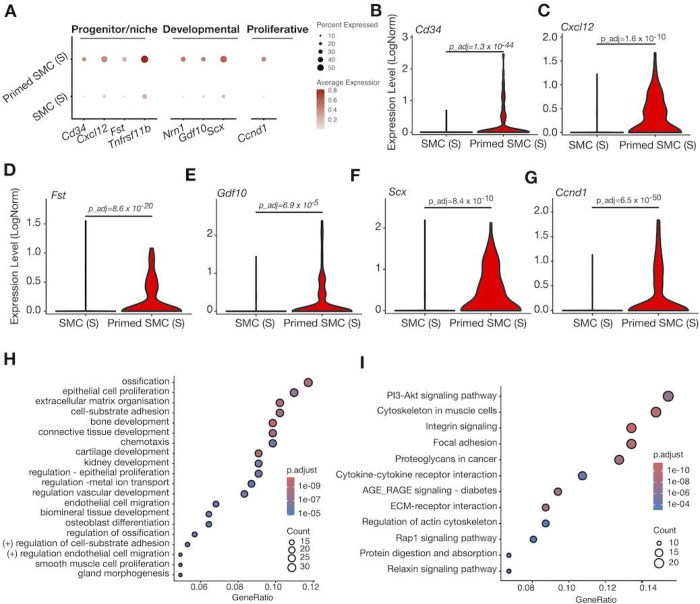
The Primed SMC progenitor-associated programme is constitutively present in the uninjured artery. (A) Dot plot of the Primed programme grouped as progenitor/niche (*Cd34, Cxcl12, Fst, Tnfrsf11b*), developmental (*Nrn1, Gdf10, Scx*) and proliferative (Ccnd1) genes (dot size, % expressing; colour, mean expression). (B–G) Violins in contractile versus Primed SMC: *Cd34* (P_adj = 1.3 × 10^−44^), *Cxcl12* (1.6 × 10^−10^), *Fst* (8.6 × 10^−20^), *Gdf10* (6.9 × 10^−5^), *Scx* (8.4 × 10^−10^), *Ccnd1* (6.5 × 10^−50^) (Wilcoxon, BH-corrected). (H,I) Over-representation analysis of genes up-regulated in Primed SMC: top GO Biological Process terms (H) and KEGG pathways (I) (dot size, gene count; colour, adjusted P; x-axis, gene ratio).

Primed SMCs displayed progenitor-niche features but lacked a classical stem-cell identity (Fig. 1C,D; Extended Data Fig. 1). They expressed a coordinated programme comprising matricellular and ECM genes (*Thbs1, Lum, Dcn* and *Col8a1*), niche-associated genes (*Cd34, Cxcl12, Tnfrsf11b* and *Fst*), developmental genes (*Nrn1, Gdf10* and *Scx*) and *Ccnd1*. This programme was enriched for ECM organisation, cell–substrate adhesion, chondro-osteogenic development and PI3K–Akt, focal-adhesion and integrin signalling, but lacked canonical stem-cell markers, including *Ly6a*/Sca1 and *Kit; Sox2, Nanog* and *Pou5f1* were undetectable. Primed SMCs nevertheless retained an attenuated contractile identity. Compared with other SMCs, they scored higher for the Primed module (*Vcam1, Timp1, Thbs1* and *Cd34*; p = 8.8 × 10^−34^) and lower for the contractile module (*Myh11, Cnn1, Myoc*d and *Carmn*; p = 2.7 × 10^−15^), while retaining reduced expression of canonical contractile genes in nearly all cells (Fig. 1E–G). Consistent with bona fide SMC identity, 78.2% of Primed SMCs retained *Myh11* expression above a stringent threshold (>3 log-normalised counts) while co-expressing the defining Primed markers and maintaining a positive module score (+0.19). By contrast, all other mural populations with Myh11 >3 lacked this signature (Fig. 1H–J).

Independent de novo re-clustering of sham cells recovered the same compartment as a high-purity orthocluster (n = 56; 92.9% Primed; r = 0.97 with integrated Primed SMCs) retaining both contractile identity and the full Primed signature (Extended Data Fig. 3), confirming it is not an integration artefact. Relative to candidate vessel-wall progenitors, *Ly6a*/Sca1 and *S100b* were primarily concentrated in endothelium and adventitial fibroblasts of sham vessels. Primed SMCs contained only a minor S100b^+^ subset (~7%) and were essentially *Ly6a*-negative (Extended Data Fig. 4A–G).

### Primed SMC population is conserved across healthy vascular beds

**Fig. 2.**
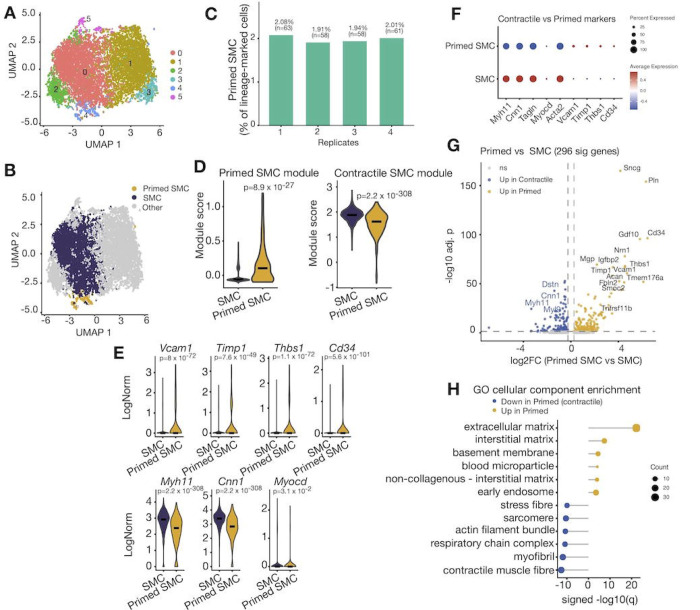
The Primed SMC compartment is conserved in the healthy aortic media of Myh11-CreERT^2^ lineage-traced mice. FACS-purified, lineage-marked aortic medial cells from across replicate libraries were clustered within the marked lineage (Harmony-integrated, resolution 0.4). (A) UMAP coloured by cluster. (B) Primed SMC (gold) and contractile SMC (cluster 0; dark blue) highlighted against remaining cells (grey). (C) Primed SMC as a percentage of lineage-marked cells in each replicate (n = 63, 58, 58, 61; 1.91–2.08) indicating consistent representation across animals. (D) Primed and contractile module scores in Primed versus contractile SMC: the Primed programme is selectively gained, the contractile module retained but modestly reduced (Wilcoxon rank-sum). (E) Violins of Primed (*Vcam1, Timp1, Thbs1, Cd34*) and contractile (*Myh11, Cnn1, Myocd*) genes (Wilcoxon, BH-corrected; bars, medians). (F) Dot plot of contractile (*Myh11, Cnn1, Tagln, Myocd, Acta2*) and Primed markers (dot size, % expressing; colour, scaled mean). (G) Volcano plot, Primed versus contractile SMC (296 significant genes; |log_2_FC| > 0.25, BH-adjusted p < 0.05): Primed-enriched (gold), contractile-enriched (blue). (H) GO cellular-component enrichment for genes up- (gold) and down-regulated (blue) in Primed SMC — extracellular-matrix/matricellular versus contractile-apparatus/oxidative terms, respectively (point size, gene count; x-axis, signed –log_10_q).

To test conservation beyond the carotid, we analysed *Myh11*-CreERT^2^ lineage-traced aortic medial scRNA-seq across four replicate libraries^10^. Clustering resolved a discrete Primed SMC population alongside contractile SMCs (Fig. 2A,B), comprising 240 lineage-marked cells at consistent frequency across replicates (1.91–2.08%; Fig. 2C). As in the carotid, contractile markers (*Myh11, Cnn1, Tagln, Acta2*) remained high but modestly reduced while the defining Primed markers (*Vcam1, Timp1, Thbs1, Cd34*) were selectively enriched (Fig. 2D–F). Genome-wide DE identified 296 distinguishing genes (matricellular/ECM regulators enriched in Primed; contractile and oxidative machinery in contractile SMC; Fig. 2G,H), demonstrating that the Primed state is conserved in healthy aortic media and reproducible across independent libraries.

### Primed SMC and a proliferating subset expand after ligation

**Extended Data Fig. 2.**
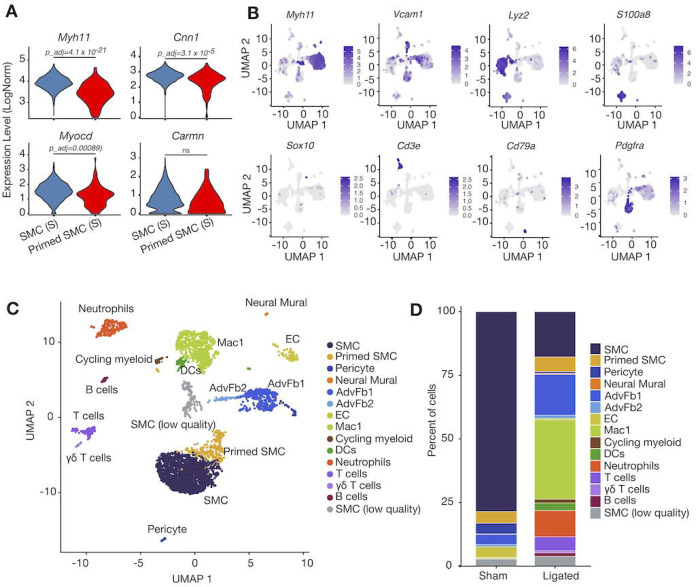
Whole-atlas markers and balanced down-sampling of sham versus ligated carotid scRNA-seq. (A) Violins of canonical contractile markers (*Myh11, Cnn1, Myocd, Carmn*) in sham SMC versus Primed SMC — retained but attenuated in Primed SMC (Wilcoxon two-sided). (B) Feature plots of compartment markers: *Myh11* (SMC), *Vcam*1 (Primed SMC), *Lyz2* and *S100a8* (innate immune), *Sox10* (neural mural/Schwann), *Cd3e* (T cell), *Cd79a* (B cell), *Pdgfra* (fibroblast). (C) UMAP after balanced down-sampling equalising Sham and Ligated cluster representation, confirming Primed SMC expansion is not a sampling artefact. (D) Composition after balanced down-sampling.

Following ligation, the contractile SMC compartment declined from 77.7% to 17.8% of cells (4.4-fold; Extended Data Fig. 2C,D), whereas Primed SMCs were the only SMC population to expand (Fig. 1A,B). This was not a capture artefact as reduced UMIs and gene detection in ligated cells were restricted to contractile SMCs and adventitial fibroblasts (contractile UMI −27%, genes −14%), while Primed SMCs were unchanged (UMI +8%, p = 0.18; genes +4%, p = 0.22; Supplementary Fig. 3), consistent with the documented apoptotic depletion of medial SMCs after ligation^39–41^ rather than under-capture of Primed cells.

Sub-clustering of the residual contractile compartment identified five sub-states, dominated by an injury-emergent stress state. However, Primed module scores remained near baseline in every contractile sub-cluster (Extended Data Fig. 5). In sham samples, Cd34 was strongly enriched in Primed SMCs relative to contractile SMCs (+6.2 log_2_FC; 25.7% versus 1.2%). Its expression remained stable during Primed SMC expansion but low across all contractile SMC sub-clusters (0–7%). These findings suggest that the expanding Primed SMC compartment retained its Cd34^+^ identity rather than recruiting Cd34-negative contractile cells.

**Fig. 3.**
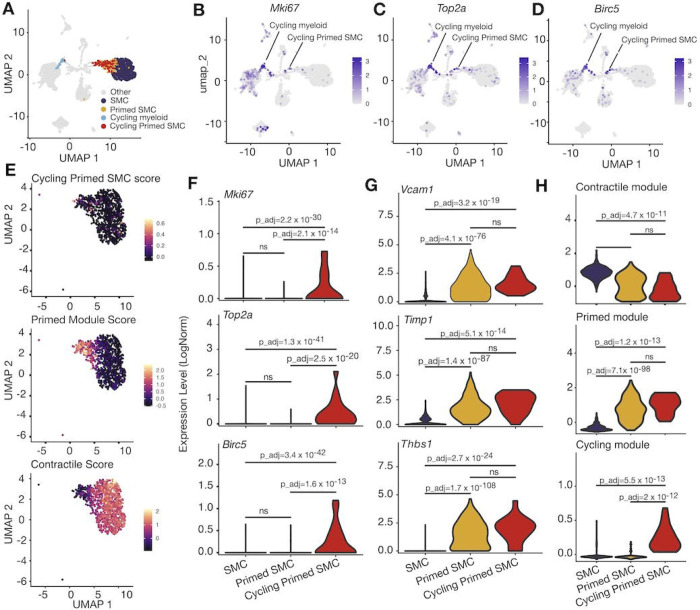
A discrete proliferating population emerges after ligation within the Primed SMC manifold. (A) UMAP of ligated cells highlighting SMC, Primed SMC, Cycling myeloid and Cycling Primed SMC (n = 20; 0.5% of ligated cells); other cells grey. (B–D) Feature plots of *Mki67* (B), *Top2a* (C) and *Birc5* (D), resolving two spatially distinct cycling foci (arrows): Cycling Primed SMC adjacent to the Primed pool, and Cycling myeloid within the macrophage compartment. (E) Feature plots of the Cycling Primed SMC, Primed and contractile module scores across the ligated SMC compartment. (F) Violins of *Mki67, Top2a* and *Birc5* in SMC, Primed SMC and Cycling Primed SMC; mitotic markers are selectively induced in the cycling subset. (G) Violins of *Vcam1, Timp*1 and *Thbs1* in the same groups; the Primed programme is equivalent in cycling and non-cycling Primed SMC. (H) Contractile, Primed and cycling module scores per group: Cycling Primed SMC retain the Primed module at non-cycling levels while selectively activating the cycling module.

A small cycling population expressing *Mki67, Top2a* and *Birc5* emerged within the SMC compartment. These cells were transcriptionally adjacent to Primed SMCs but distinct from cycling myeloid cells (Fig. 3A–D). In ligated vessels, this cycling Primed SMC subset (n = 20) selectively upregulated mitotic markers while retaining the core Primed programme (*Vcam1, Timp1* and *Thbs1*) and its module scores were unchanged relative to non-cycling Primed SMCs (Fig. 3F–H). These findings support proliferation within the pre-existing Primed SMC compartment rather than the acquisition of a new fate by contractile SMCs.

Ligation induced *Ly6a* expression in Primed SMCs (p_adj = 7.4 × 10^−8^) but had no effect on *S100b* and reduced *Myh11* expression (p_adj < 2.2 × 10^−308^; Extended Data Fig. 4H–J).

### Pre-existing Primed SMC are the dominant inferred source of expanded lesion-associated SMC populations

**Fig. 4.**
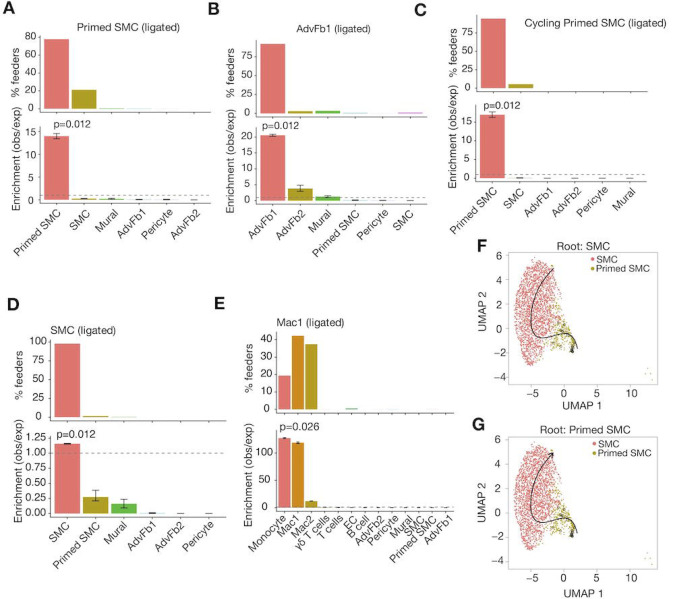
Primed SMC are the dominant inferred transcriptional source of the expanded lesion SMC pool after carotid ligation. (A–E) Forward k-nearest-neighbour (kNN) feeder analysis. Each ligated target was mapped to its nearest sham neighbours (k = 15; 20 Harmony dimensions); sources are scored as mean neighbour fraction (top, % feeders) and as enrichment over background frequency (bottom, observed/expected). Bars, point estimate; error bars, 95% bootstrap CI (n = 50); dashed line, obs/exp = 1; P, permutation test (n = 500) for the top source, Bonferroni-corrected across reference clusters. References were the mesenchymal compartment (SMC, Primed SMC, AdvFb1, AdvFb2, Pericyte, Mural) in A–D and the full sham reference in E. (A) Ligated Primed SMC self-source from sham Primed SMC (~78%; ~14×), with contractile SMC depleted (0.25×). (B) Ligated AdvFb1 self-source from sham AdvFb1 (~92%; ~21×). (C) Cycling Primed SMC (S/G2M plus positive Mki67/Top2a/Birc5 module; n = 20) self-source from sham Primed SMC (~95%; ~17×). (D) Ligated SMC self-source from sham SMC (98%), with Primed SMC depleted (0.27×); the modest self-enrichment (1.16×) reflects SMC dominating the reference, so the directional signal is near-complete self-feeding plus Primed depletion. (E) Specificity control: ligated Mac1 source exclusively from myeloid populations (>100×), with SMC and Primed SMC contributions indistinguishable from zero. (F,G) Dual-rooted pseudotime (Slingshot) over the contractile–Primed axis, rooted at contractile SMC (F) and Primed SMC (G): the single fitted lineage is identical in topology and reverses with the root, indicating one contractile–Primed continuum while remaining agnostic to direction; open circles, cycling Primed SMC (n = 20).

To distinguish autonomous expansion from recruitment of contractile SMCs, we used k-nearest-neighbour mapping against the sham reference to infer the transcriptional source of each ligated population and quantified source enrichment. Ligated Primed SMCs mapped overwhelmingly to the sham Primed pool (77.9%; 14.0-fold enrichment), whereas contractile SMCs were depleted as a potential source (0.25-fold; Fig. 4A). The cycling Primed SMC subset mapped even more strongly to sham Primed SMCs (94.7%; 17.0-fold enrichment; Fig. 4A,C), placing the proliferative response within the pre-existing Primed SMC compartment. The other populations showed the same lineage-restricted architecture: ligated AdvFb1 cells mapped almost exclusively to sham AdvFb1 cells (92%; ~21-fold enrichment; Fig. 4B), while ligated contractile SMCs mapped predominantly to sham contractile SMCs (98%; Fig. 4D), with Primed SMCs depleted as a source (0.27-fold). As a specificity control, ligated Mac1 cells mapped exclusively to myeloid populations, with SMC contributions indistinguishable from zero (Fig. 4E).

Dual-rooted pseudotime analysis with Slingshot placed contractile and Primed SMCs along a single continuous axis, but the inferred direction reversed completely depending on the selected root (Fig. 4F,G). This finding confirmed continuity between the states while remaining agnostic about directionality, which was instead resolved by the feeder analysis. The reciprocal sham-to-ligated analysis reproduced the same pattern (Extended Data Fig. 6), with sham contractile SMCs depleted as a source of ligated Primed SMCs (0.22-fold). However, because nearest-neighbour sourcing measures transcriptional similarity rather than lineage, it cannot by itself exclude convergence of modulated contractile SMCs on the Primed state. We therefore examined two genetic lineage-tracing models of atherosclerosis, in which temporal kinetics could distinguish autonomous expansion from phenotypic conversion.

### Autonomous expansion of the Primed SMC pool dominates lesion cellularity in atherosclerosis

**Fig. 5.**
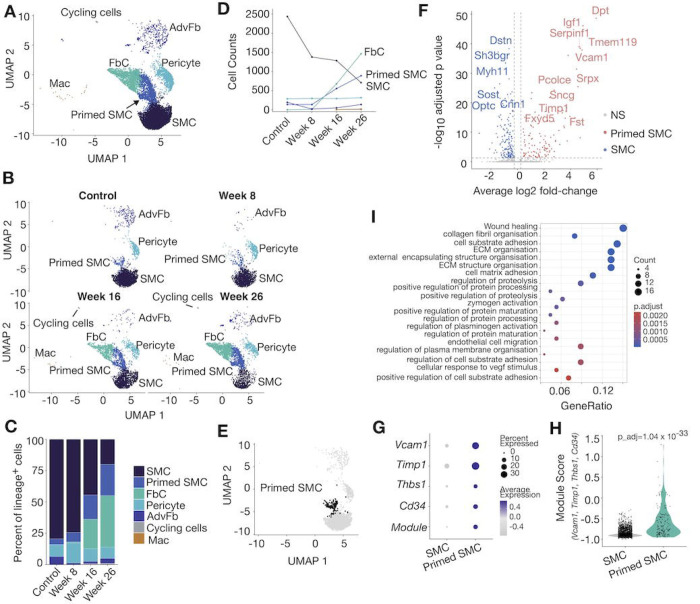
A pre-existing Primed SMC pool expands progressively in LDLR atherosclerosis. LDLR^−/−^ Myh11-CreERT^2^ ZsGreen1-WPRE scRNA-seq; lineage-positive cells retained at ZsGreen1-WPRE > 3.17 log-norm (n = 11,362). (A) Harmony-integrated UMAP showing SMC, Primed SMC, FbC, AdvFb, Pericyte/Neural Mural, Cycling cells and Mac. (B) UMAP per timepoint (Control, Weeks 8/16/26), showing progressive emergence of disease-expanded Primed SMC, FbC and Cycling cells. (C) Stacked-bar composition of lineage-positive cells across timepoints. (D) Cell counts per timepoint: SMC contract monotonically (2,435 → 1,378 → 1,286 → 710; 3.4×); Primed SMC expand 6.3× (141 → 140 → 556 → 890), chiefly between Weeks 8 and 16; FbC emerge at Week 16 and become the largest lineage-positive compartment by Week 26 (3 → 15 → 679 → 1,464). (E) UMAP highlighting Control Primed SMC. (F) Volcano plot, Control Primed SMC versus Control SMC (Wilcoxon two-sided, BH-corrected); selected genes labelled. (G) Dot plot of Primed module-gene expression in Control SMC versus Control Primed SMC. (H) Primed SMC module score in Control SMC (n = 2,435) versus Control Primed SMC (n = 141); p_adj = 1.04 × 10^−33^ (Wilcoxon, BH-corrected). (I) GO Biological Process over-representation among genes up-regulated in Control Primed SMC, recapitulating the sham-carotid architecture.

To test whether Primed SMCs act as the autonomous source of *Myh11*-lineage lesion populations, we analysed two *Myh11*-CreERT^2^ ZsGreen1-WPRE lineage-traced datasets spanning Control, Week 8, Week 16 and late disease (Week 26 LDLR^−/−^; Week 22 ApoE^−/−^)^23^. A ZsGreen1-WPRE > 3.17 threshold, restricted to SMC-lineage compartments, retained 11,362 LDLR and 9,015 ApoE lineage-positive cells. Harmony clustering resolved contractile SMC, a discrete Primed SMC cluster, adventitial fibroblasts, pericytes, a disease-emergent fibromyocyte/fibrochondrocyte (FbC) compartment, a cycling cluster and a minor lineage-positive macrophage-like population (Fig. 5A; Supplementary Table 2).

The lineage-positive compartment underwent marked remodelling during disease progression (Fig. 5B–D). Contractile SMCs declined from 79.5% of lineage-positive cells at Control to 19.9% at Week 26 (2,435 to 710 cells; 3.4-fold decrease). In contrast, Primed SMCs increased from 4.6% to 25.0% (141 to 890 cells; 6.3-fold increase). FbC were almost absent at Control and Week 8 (3 and 15 cells, respectively), but emerged by Week 16 (679 cells; 23.5%) and became the largest lineage-positive population by Week 26 (1,464 cells; 41.1%). Together, Primed SMCs, FbC and cycling cells increased from 4.7% to 66.8% of lineage-positive cells. The temporal kinetics argue against widespread conversion of contractile to Primed SMCs. By Week 8, the SMC compartment had already contracted by 40%, driven by a 43% loss of contractile SMCs, whereas Primed SMC numbers remained unchanged (141 versus 140 cells) and FbC remained rare (15 cells; Fig. 5D; Supplementary Fig. 4B). Thus, contractile SMC loss preceded Primed SMC and FbC expansion.

The overall lineage-positive fraction remained stable throughout disease progression (Supplementary Fig. 4E), arguing against reporter silencing. The early decline in contractile SMCs is consistent with the documented apoptosis of medial SMCs in atherosclerosis^41–43^. The subsequent recovery of the broader SMC-lineage compartment reflected Primed SMC expansion and FbC emergence rather than restoration of the contractile SMC population. Comparison of control Primed SMCs with control contractile SMCs confirmed preservation of the homeostatic Primed SMC identity (Fig. 5E–H). Functional enrichment in control Primed SMCs was dominated by ECM and collagen-fibril organisation, cell–substrate and cell–matrix adhesion, and wound healing. Additional enriched pathways involved proteolysis and plasminogen activation, as well as angiogenic processes such as endothelial migration and responses to VEGF (Fig. 5I).

### Temporal feeder analysis supports autonomous expansion of Primed SMC during atherosclerosis

**Fig. 6.**
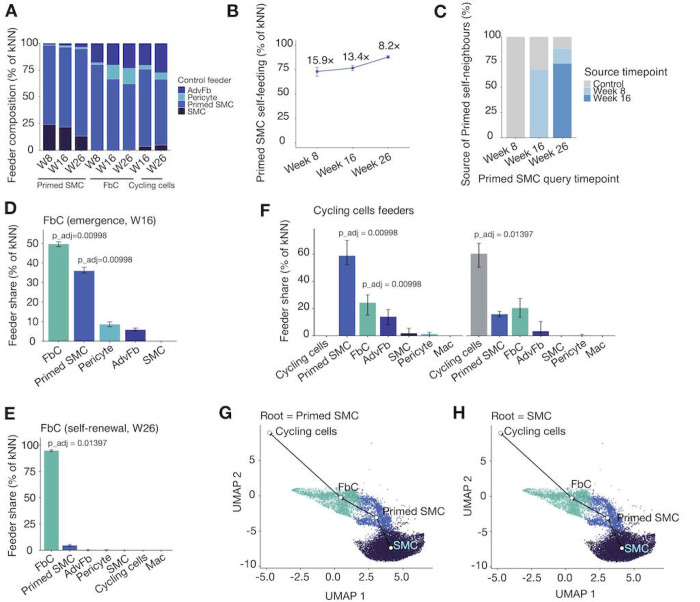
Temporal kNN feeder-source analysis identifies the pre-existing Primed SMC pool as the autonomous source of disease-expanded SMC populations in LDLR atherosclerosis. Forward kNN feeder-source analysis of lineage-positive (ZsGreen1-WPRE > 3.17) cells across Control and Weeks 8/16/26 (20 Harmony dimensions, k = 15). Error bars, bootstrap 95% CI (50 iterations); P, 500-permutation test, Bonferroni-corrected across reference clusters. Because the corrected floor is ≈0.010 at five clusters (Weeks 8/16) and ≈0.014 at seven (Week 26), the strongest Week-26 associations read * (p_adj < 0.05) rather than ** — a reflection of permutation resolution, not weaker enrichment. (A) Composition of each query's *baseline* neighbours (contractile SMC, Primed SMC, pericyte, AdvFb), isolating the pre-existing source: Primed SMC dominate across all three disease populations and timepoints (62–81%); contractile SMC contribute only to the Primed query and decline (23.9% → 13.3%), with AdvFb the secondary input to FbC and Cycling cells. (B) Primed SMC self-feeding rises across timepoints — 73.1% (15.9×), 76.7% (13.4×), 88.0% (8.2×) at Weeks 8/16/26 (p_adj ≤ 0.014) — while contractile SMC are progressively depleted as a feeder (0.30× → 0.21× → 0.07×); the falling fold-enrichment reflects expansion of the Primed fraction within the cumulative reference, not reduced self-mapping. (C) Source-timepoint decomposition: each disease Primed pool is fed mainly by the immediately preceding one (67.8% from Week 8 at Week 16; 73.6% from Week 16 at Week 26), the residual Control input declining (32.2% → 11.6%) — stepwise forward propagation. (D) FbC at emergence (Week 16): besides self-neighbours (49.6%; 135.5×), the dominant baseline source is Primed SMC (35.9%; 6.3×; both p_adj = 0.00998), with negligible contractile SMC input. (E) FbC at Week 26: maintained almost entirely by self-renewal (94.8%; 10.6×; p_adj = 0.014), 98.4% mapping to the Week-16 FbC pool. (F) Cycling cells: at first detection (Week 16) fed mainly by Primed SMC (58.8%; 10.3×) with a secondary FbC input (24.2%; 66.2×; both p_adj = 0.00998) and no contractile SMC input; by Week 26 predominantly self-renewing (60.3%; 427.8×; p_adj = 0.014). The Week-26 Cycling population is small (n = 22) and k-sensitive (Supplementary Fig. 5C) and is read as a small-k tendency. (G,H) Dual-rooted Slingshot over the SMC-fate compartment (contractile SMC, Primed SMC, FbC, Cycling cells). Rooting at Primed SMC (G) resolves two lineages (Primed SMC → FbC → Cycling cells, and a branch to contractile SMC); rooting at contractile SMC (H) forces a single linear trajectory. Slingshot establishes topology only; direction is adjudicated by the feeder analysis (B–F).

To test whether Primed SMCs expanded autonomously, we performed temporal kNN feeder analysis by querying each time point against all preceding time points, with bootstrap resampling and permutation testing (Fig. 6A). Primed self-feeding dominated throughout, increasing from 73.1% at Week 8 to 76.7% at Week 16 and 88.0% at Week 26 (permutation-adjusted P ≤ 0.014; Fig. 6B). The corresponding decline in enrichment (15.9-fold to 13.4-fold to 8.2-fold) reflected the increasing proportion of Primed SMCs in the reference population rather than reduced specificity. Despite their predominance at baseline, contractile SMCs remained below background as a source of Primed SMCs (0.30-fold to 0.21-fold to 0.07-fold). At Week 16, when FbCs emerged, their largest baseline-derived contribution came from Primed SMCs (35.9%; 6.3-fold), with negligible input from contractile SMCs. Cycling cells were similarly derived predominantly from Primed SMCs (58.8%), with no detectable contractile SMC contribution. By Week 26, both FbCs and cycling cells were almost entirely self-renewing (Fig. 6C–F; Supplementary Fig. 5C).

Rooted-tree analysis of the SMC-fate compartment placed contractile SMCs at a terminal node and Primed SMCs at an internal node. Using contractile SMCs as the root forced a single linear trajectory (SMC → Primed → FbC → Cycling; Fig. 6H), whereas rooting the tree in Primed SMCs resolved two distinct lineages (Primed → FbC → Cycling and Primed → SMC; Fig. 6G). Thus, the topology does not require a contractile SMC root and is compatible with Primed SMCs occupying the apex.

**Fig. 7.**
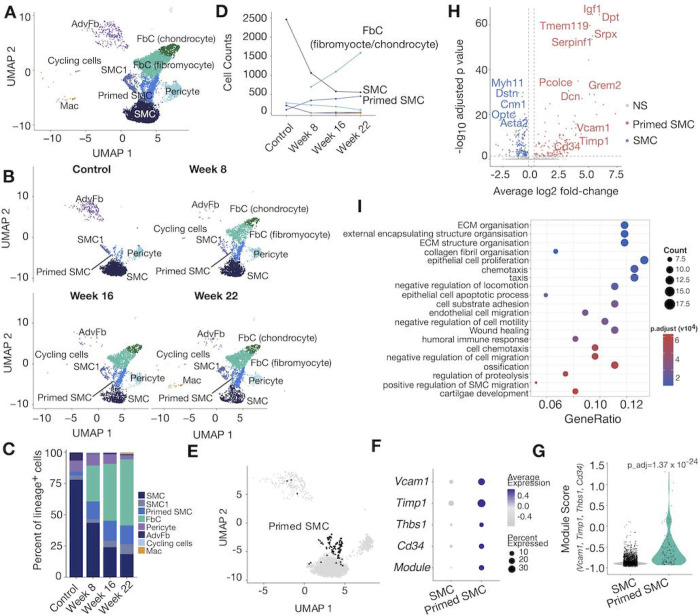
ApoE atherosclerosis recapitulates the autonomous-expansion architecture with distinct kinetics. Myh11-CreERT^2^;ZsGreen1-WPRE lineage-traced ApoE^−^/^−^ aortic cells across Control and Weeks 8/16/22; lineage-positive cells (ZsGreen1-WPRE > 3.17; n = 9,015) clustered de novo, independently of LDLR. ApoE resolves a major contractile SMC cluster and a minor SMC1 subset absent from LDLR; FbC denotes the fibromyocyte–fibrochondrocyte compartment. (A) UMAP by cell type. (B) Same UMAP by timepoint. (C) Lineage-positive composition across timepoints: contractile SMC contract while Primed SMC and FbC expand reciprocally. (D) Per-timepoint lineage-positive counts (contractile SMC, Primed SMC, FbC labelled); FbC dominates by Week 22. (E) UMAP highlighting Control Primed SMC. (F) Dot plot of Primed markers and composite module in Control contractile versus Primed SMC (dot size, % expressing; colour, scaled mean). (G) Primed module score, contractile versus Primed SMC; p_adj = 1.37 × 10^−24^ (Wilcoxon rank-sum, Bonferroni-corrected). (H) Volcano plot (Primed versus contractile SMC, Control): Primed-enriched (red) include Igf1, Dpt, Tmem119, Srpx, Serpinf1, Pcolce, Dcn, Grem2, Vcam1, Timp1, Cd34; contractile-enriched (blue) include Myh11, Acta2, Cnn1, Dstn, Optc (dashed lines, |avg log_2_FC| = 0.25 and p_adj = 0.05). (I) GO Biological Process over-representation among Primed-enriched genes — extracellular-matrix organisation, cell–substrate adhesion, vascular remodelling, ossification and cartilage development (dot size, gene count; colour, adjusted P).

The independent ApoE−/− model (Control to Week 22) reproduced a similar architecture (Fig. 7A–I; Supplementary Table 3). It recapitulated Primed SMC expansion and the emergence and dominance of FbC that retained the Primed SMC programme (p_adj = 1.37 × 10^−24^). Other shared features included enrichment of ECM, adhesion, vascular-remodelling and ossification pathways; dominant Primed self-feeding, which declined from 23.6-fold at Week 8 to 8.5-fold at Week 22; contractile depletion; joint contributions from Primed SMC and AdvFb cells to FbC; and equivalent dual-rooting (Extended Data Fig. 7). Cell-cycle regression showed that the apparent contractile contribution to cycling cells was an embedding artefact (Supplementary Fig. 5A,B,D).

In both models, contractile sub-clustering identified multiple injury-associated stress and activation states. However, the defining Primed markers remained restricted to Primed SMCs and were not acquired by any contractile sub-cluster (Extended Data Fig. 8), demonstrating that autonomous Primed SMC expansion is conserved across both genetic models.

### *Ly6a* and *S100b* mark the pre-existing Primed SMC pool and its disease-induced activation

Cross-cohort comparison at baseline using BH-corrected Wilcoxon tests showed that *S100b* and *Ly6a* mark complementary components of the Primed programme (Extended Data Fig. 9A,B). *S100b* was enriched in Primed SMCs across all four cohorts, reaching significance in the sham carotid and aortic medial datasets, with borderline enrichment in LDLR and a concordant trend in ApoE. By contrast, *Ly6a* enrichment was largely restricted to the aortic media, recapitulating the canonical Ly6a/Sca1^+^ aortic Primed state^10^ and indicating that *Ly6a* is not a universal marker of homeostatic Primed SMCs. During disease progression*, Ly6a* was strongly induced in Primed SMCs in both models, reaching levels well above those observed during homeostasis, whereas *S100b* remained relatively stable (Extended Data Fig. 9C,E). FbC expressed both markers from their emergence and maintained strong *Ly6a* expression (Extended Data Fig. 9D,F), supporting molecular continuity between homeostatic Primed SMCs and lesion-emergent FbC. Thus, neither marker alone defines the Primed state; instead, they represent overlapping subprogrammes within a broader conserved identity.

### Primed SMC carry a conserved *Cd34*^+^ progenitor-like programme as a single poised state

To define the intrinsic identity of Primed SMCs, we compared baseline Primed and contractile SMCs across four cohorts. Every dataset revealed a conserved programme comprising a niche/progenitor module (*Cd34, Tnfrsf11b, Phlda1, Fst* and *Eng*) and a matricellular module (*Vcam1, Thbs1, Timp1, Mgp, Lum* and *Dcn*; Extended Data Fig. 10E). This programme was progenitor-associated rather than pluripotent: canonical stem-cell markers (*Sox2, Nanog, Pou5f1* and *Kit*) were not enriched, *Ly6a* enrichment was confined to the aortic media, and phenotypic-switching factors (*Klf4, Sox9* and *Nes)* were not enriched. *Runx2 i*ncreased only in the carotid and aortic medial datasets, consistent with lineage-specific chondrogenic priming. The core contractile programme nevertheless remained robustly expressed. The niche/progenitor and matricellular modules defined a single coupled state rather than distinct subpopulations. Within Primed SMCs, their scores were positively correlated across all four cohorts (Spearman ρ = +0.13 to +0.52), and both tended to correlate inversely with the contractile programme, the opposite of that predicted for two separate subpopulations (Extended Data Fig. 10A–D). Hartigan’s dip test found no evidence of bimodality (all p > 0.23), and sub-clustering did not identify progenitor-pure or contractile-pure populations. Primed SMCs therefore represent a single continuous Cd34^+^ progenitor-like state that co-expresses niche/progenitor and matricellular programmes on a retained contractile background.

### A pan-vascular Primed SMC identity with bed-predominant axes

**Fig. 8.**
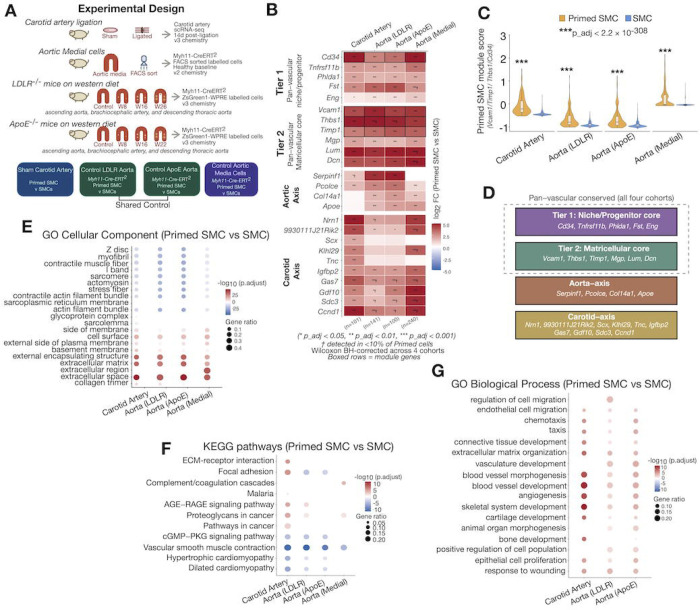
A conserved, progenitor-like Primed SMC identity is shared across vascular beds. (A) Four baseline comparisons spanning three independent biological baselines (sham carotid artery, FACS-purified healthy aortic media, and a single atherosclerosis Control re-analysed within both the LDLR and ApoE pipelines). Primed versus contractile SMC were compared at baseline in four independent cohorts: sham carotid artery (14 d, v3); FACS-sorted Myh11-CreERT2 lineage^+^ healthy aortic media (v2); and control ascending aorta from LDLR^−^/^−^ and ApoE^−^/^−^ mice on Western diet (Myh11-CreERT2, ZsGreen1-WPRE lineage^+^, v3). Each cohort was processed natively through the same pipeline. (B) Tiered log_2_ fold-change heatmap (Primed versus contractile SMC) for 25 signature genes (colour capped at ±5; all genes tested without a detection filter). Asterisks, Wilcoxon p_adj (BH across the full four-cohort matrix; *<0.05, **<0.01, ***<0.001); †, gene detected in <10% of Primed SMC (less stable effect size); boxed rows, the four-gene module scored in C. Genes group into a pan-vascular niche/progenitor core (Tier 1) and matricellular core (Tier 2), an aorta-predominant axis (Tier 3) and a carotid-predominant axis (Tier 4); axes denote predominant, not exclusive, enrichment. n (Primed SMC) per cohort below each column. (C) Primed SMC module score (Vcam1, Timp1, Thbs1, Cd34) in Primed versus contractile SMC per cohort, shared y-axis (box, IQR; whiskers, 1.5× IQR; Wilcoxon, BH-corrected; all p_adj < 2.2 × 10^−308^). Scores are background-relative; the conserved result is the within-cohort Primed > SMC enrichment. (D) Summary: two conserved pan-vascular cores (dashed box, all four cohorts) and two bed-specific axes. (E–G) Over-representation analysis of Primed-versus-contractile DE genes (Wilcoxon p_adj < 0.05, |log_2_FC| > 0.25; min.pct = 0.10); dot colour, signed −log_10_p_adj (red, enriched in Primed; blue, depleted); dot size, gene ratio. (E) GO Cellular Component, all four cohorts. (F) KEGG pathways, all four cohorts. (G) GO Biological Process, carotid/LDLR/ApoE (aortic media omitted).

We compared baseline Primed and contractile SMCs across all four cohorts using a common analytical pipeline (Fig. 8A). The prespecified four-gene Primed module scored higher in Primed SMCs in every cohort (all p_adj < 2.2 × 10^−308^), and each constituent gene was individually enriched, confirming the module’s out-of-sample validity (Fig. 8C). Differential-expression analysis identified a tiered architecture comprising two conserved cores—niche/progenitor and matricellular—and two vascular-bed-predominant axes: an aortic axis (*Serpinf1, Pcolce, Col14a1* and *Apoe*) and a carotid axis (*Nrn1, Scx, Tnc, Igfbp2, Gas7, Gdf10* and *Ccnd1*; Fig. 8B,D).

Using a uniform detection threshold of counts >0, which provides a conservative lower bound on prevalence, Cd34 was detected in 10.5–25.7% of Primed SMCs versus 0.1–2.1% of contractile SMCs. *Vcam1* was detected in 21.5–51.5% versus 2.1–6.5%, whereas *S100b* was detected in only 0.8–6.9% versus 0.1–1.8%. *Cd34* showed the greatest specificity, with a 6- to 105-fold difference between the populations. In an independent acute carotid-injury dataset collected 3 days after ligation^28^, Cd34^+^ cells already constituted 16.1% of the Primed compartment at baseline and did not increase acutely despite expansion of the compartment. This pattern is opposite to that expected if Cd34 were acquired de novo. Thus, *Cd34* and *Vcam1* mark a substantial and specific fraction of the pre-existing Primed compartment.

Functional enrichment across all four cohorts confirmed a contractile-low, matricellular-high architecture (Fig. 8E–G). Primed SMCs were depleted of sarcomeric machinery and of vascular smooth-muscle contraction and cGMP–PKG signalling pathways, but enriched for ECM–receptor, proteoglycan and AGE–RAGE pathways. Four broader programmes were already active during homeostasis: migration and chemotaxis, ECM organisation and adhesion, vasculature development, and skeletal and cartilage development. The skeletal/cartilage programme was strongest in carotid Primed SMCs, foreshadowing the fibrochondrocyte fate. An integration-free metric based on the similarity of cohort-level fold changes independently confirmed Primed SMCs as the same cell type across cohorts (Supplementary Fig. 6).

Two additional analyses that were independent of root selection and pseudotime—partition-based graph abstraction and cross-time-point temporal flow—placed Primed SMCs as the sole conduit between contractile and disease-expanded states. Both inferred directionality from Primed SMCs despite the numerical predominance of contractile SMCs at baseline (Supplementary Fig. 7). Feeder-source analysis, pseudotime and graph-abstraction/temporal-flow analysis rely on distinct assumptions yet converged on the same architecture, identifying the homeostatic Primed compartment as the dominant inferred ancestral source of lesion-associated SMC populations.

### The lesion fibromyocyte and fibrochondrocyte states are a continuation of the Primed SMC programme

**Fig. 9.**
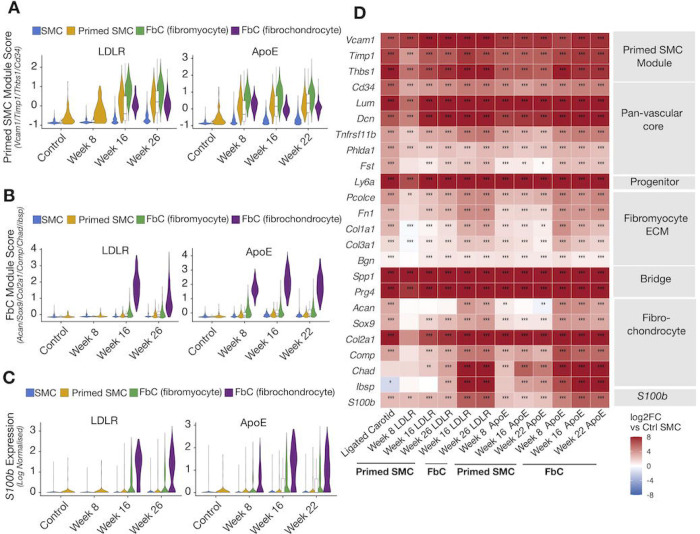
Primed SMC identity is preserved across the Primed SMC → fibrochondrocyte trajectory in both atherosclerosis cohorts. Native, per-cohort annotation of contractile SMC, Primed SMC and FbC (fibromyocyte/fibrochondrocyte); the LDLR^−^/^−^ fibrochondrocyte was resolved by sub-clustering the FbC compartment, the ApoE^−^/^−^ fibrochondrocyte is a native cluster. (A–C) Per-cell distributions across disease timepoints — LDLR^−^/^−^ (Control/Weeks 8/16/26) and ApoE^−^/^−^ (Control/Weeks 8/16/22): (A) Primed module score (*Vcam1/Timp1/Thbs1/Cd34*); (B) fibrochondrocyte module score (*Acan/Sox9/Col2a1/Comp/Chad/Ibsp*); (C) *S100b* (log-normalised). AddModuleScore; box, IQR; whiskers, 1.5× IQR. (D) Heatmap of 24 marker genes across 13 columns (Ligated Carotid, then Primed SMC and FbC at each disease timepoint per cohort), grouped as Primed module (*Vcam1, Timp1, Thbs1, Cd34*), pan-vascular core (Lum, Dcn, Tnfrsf11b, Phlda1, Fst), progenitor *(Ly6a*), fibromyocyte ECM (*Pcolce, Fn1, Col1a1, Col3a1, Bgn*), secreted bridge (*Spp1, Prg4*), fibrochondrocyte (*Acan, Sox9, Col2a1, Comp, Chad, Ibsp*) and *S100*b. Colour, log_2_ fold change versus the cohorťs baseline contractile SMC (capped at ±8); stars, Wilcoxon BH-adjusted p (*<0.05, **<0.01, ***<0.001).

Feeder and trajectory analyses identified Primed SMCs as the dominant inferred source of lesion-associated FbC. We therefore asked whether FbC retain the Primed programme or converge on an unrelated state. The Primed module (*Vcam1, Timp1, Thbs1* and *Cd34*) remained low in contractile SMCs at every time point in both models but was elevated in Primed SMCs, fibromyocytes and fibrochondrocytes (Fig. 9A). Module expression was highest in fibromyocytes and, although attenuated in terminal fibrochondrocytes, never returned to the contractile baseline. By contrast, the chondrogenic programme (*Acan, Sox9, Col2a1, Comp, Chad* and *Ibsp)* was restricted to fibrochondrocytes (Fig. 9B). This programme emerged during disease—at Week 16 in LDLR−/− mice and Week 8 in ApoE−/− mice—and localized to a discrete cluster in ApoE−/− and within the LDLR−/− FbC compartment. Four signatures were shared by Primed SMCs and FbC across both models and the carotid reference: the Primed surface module, a pan-vascular core, *Ly6a* and a secreted bridge programme defined by *Spp1* and *Prg4* (Fig. 9D). In contrast, fibromyocyte ECM genes and the terminal chondrogenic programme appeared only after lesion formation. Lesion-associated FbC states therefore represent progressive elaborations of a pre-existing Primed programme rather than unrelated endpoints.

### A Primed-like SMC state pre-exists in the healthy human aorta and shares a conserved transcriptional core with mouse

**Figure 10.**
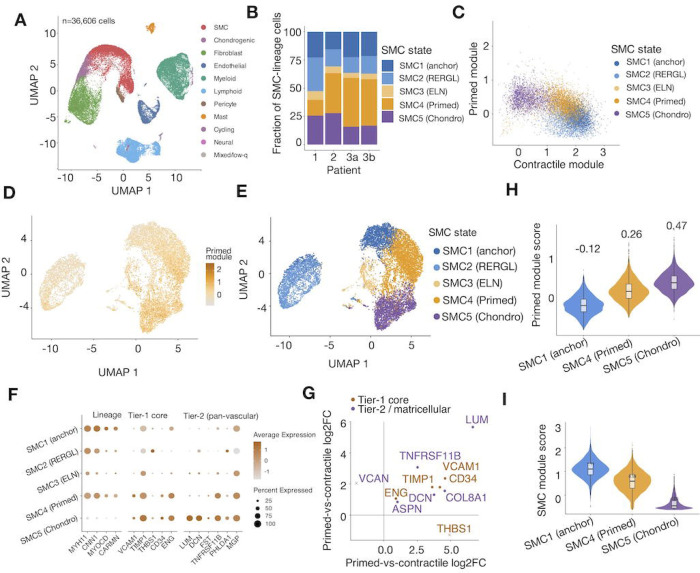
A Primed-like SMC state pre-exists in healthy human aorta. Aortic samples of a human vessel atlas^27^ processed de novo through the mouse pipeline. SMC states: SMC1 (contractile anchor), SMC2 (RERGL^+^ contractile subset), SMC3 (ELN^+^, elastin-high), SMC4 (Primed) and SMC5 (chondrogenic). (A) Lineage atlas (integrated UMAP). (B) SMC-state composition across the four aortic samples. (C) Per-cell Primed- versus contractile-module score, coloured by SMC state. (D) Primed-module score (*VCAM1, TIMP1, THBS1, CD34*) on the SMC embedding. (E) SMC embedding coloured by state. (F) Lineage, Tier-1 core and Tier-2 pan-vascular marker expression by SMC state (dot size, % expressing; colour, mean expression). (G) Cross-species directional concordance of the eleven Tier-1/Tier-2 genes (human SMC4 vs SMC1 against the mouse Primed-vs-contractile contrast); ×, discordant. (H) Primed-module score for SMC1, SMC4 and SMC5. (I) SMC contractile-identity-module score (*MYH11, CNN1, MYOCD, CARMN*) for the same three states.

To investigate whether humans possess an equivalent population, we reanalysed the aortic compartment of a human vessel atlas^27^ using the same pipeline (36,606 cells from four samples and three donors; Fig. 10A). The SMC compartment contained a contractile reference population (SMC1; 2,098 cells, 99.5% *MYH11*^+^) and a reproducible Primed-like population (SMC4; 3,602 cells, 97.6% MYH11^+^; Fig. 10B,E). SMC4 was positioned closer to the Primed pole: it scored higher than SMC1 for the Primed module (Fig. 10H) and was displaced by +0.84 along the Primed–contractile axis (−1.38 versus −2.22; Wilcoxon p < 2.2 × 10^−308^). Across the SMC compartment, Primed and contractile module scores were inversely correlated (Spearman ρ = −0.54; Fig. 10C). Relative to SMC1, SMC4 upregulated *VCAM1, TIMP1* and *CD34*, together with the matricellular genes *LUM, TNFRSF11B, COL8A1* and *ASPN* (Fig. 10F). A contractile-identity module comprising *MYH11, CNN1, MYOCD* and *CARMN* was highest in SMC1, retained in SMC4 and sharply reduced in the chondrogenic SMC5 population (Fig. 10I), consistent with the corresponding *MYH11*^+^ fractions of 99.5%, 97.6% and 34.5%. Thus, SMC4 represents Priming within a retained SMC identity, whereas SMC5 reflects loss of this identity during progression toward an osteochondrogenic fate. We then tested directional agreement between human and murine Primed-versus-contractile contrasts across three non-injury aortic datasets (Fig. 10G). Nine of eleven Tier-1/Tier-2 genes showed the same direction of change in all three datasets (sign test p = 0.033). The two exceptions*, THBS1* and *VCAN*, diverged consistently, suggesting species-specific variation within an otherwise conserved Primed core.

## Discussion

The principal finding of the current study is that a rare homeostatic progenitor-like Primed SMC compartment is identified consistently as the dominant inferred ancestral compartment underlying lesion-associated SMC populations across both vascular injury and atherosclerosis. This derives from convergence of multiple orthogonal analyses rather than any single framework, supporting a model in which lesion-associated SMC arise predominantly through expansion and diversification of a pre-existing Primed compartment, not widespread phenotypic switching of the contractile medial majority^1,6,7^.

The Primed state was conserved across four baseline cohorts by two orthogonal approaches — a discriminating-marker analysis after integration and an integration-free CFS metric — establishing it as pre-existing rather than lesion-induced. The genetically lineage-marked aortic media provided the clearest example. SMCs carrying a matricellular programme prominent enough to resemble adventitial fibroblasts at the whole-transcriptome level, yet retaining a conserved SMC marker core. Against a conserved contractile-low background (sarcomeric machinery reciprocally lost despite preserved *Myh11/Cnn1/Myocd/Carmn*), the positive Primed programme couples a matricellular signature with a chondrogenic-poised developmental signature latent at homeostasis and consistent with the later fibrochondrocyte fate. This compartment is also present at baseline in the non-diseased human aorta^27^ displaced along the same Primed–contractile axis with directional concordance across three independent non-injury datasets (nine of eleven Tier-1/Tier-2 genes; *THBS1* and *VCAN* the species-specific exceptions) — a baseline feature of medial SMC that predates disease in both species.

Compositional shifts during disease spanning contraction of the contractile SMC compartment accompanied by Primed SMC expansion are compatible with both phenotypic switching^44,45^ and autonomous Primed expansion occurring alongside contractile SMC loss. Because these models make opposing predictions about feeder-source identity, we tested them directly. In both atherosclerosis cohorts, the homeostatic Primed compartment was the predominant inferred source of the disease-expanded Primed population at every stage and of FbC and cycling cells at their emergence. These lesion-associated compartments subsequently became self-renewing, whereas contractile SMCs were consistently depleted as a feeder source (enrichment ≪1 at k = 15). These findings were robust across neighbourhood sizes and after cell-cycle regression. Sub-state analysis further indicated that apparent turnover within the contractile compartment reflected internal drift rather than conversion to the Primed state. Moreover, two analyses independent of both root selection and pseudotime (graph abstraction and temporal flow) identified Primed SMCs as the sole conduit between contractile and disease-expanded states, with directionality originating from Primed SMCs. The convergence of methods based on distinct assumptions makes a method-specific artefact unlikely.

Disease-expanded SMC populations have previously been described as fibromyocytes^46^ SEM^23,47^ Lgals3^+^ pioneer^48^ and modulated or intermediate continuum states^49–51^. Each overlaps with the disease-expanded Primed population and shares a matricellular core defined by *Vcam1, Timp1* and *Thbs1*, but carries different marker overlays, including *Ly6a, Tcf21* and *Lgals3* that are not uniformly co-expressed. In these studies, however, transition direction was generally determined by the inference method. Trajectories were rooted in the numerically dominant contractile population, constraining the analysis to recover contractile-to-modulated transitions and preventing it from distinguishing such transitions from autonomous expansion of a minority population. When applied across lineage-resolved cohorts, the feeder-source framework instead identified the homeostatic Primed pool as the dominant source. This finding recasts the convergent observations from previous studies as consistent with autonomous expansion of a pre-existing compartment.

Ding and colleagues attributed a Vcam1^+^ proinflammatory SMC population to phenotypic switching on the basis of pseudotemporal ordering^28^. Our reanalysis of their 3-day ligation dataset recovered this population within the same conserved matricellular programme. However, our feeder-source analysis of their data inferred an origin in the pre-existing Primed compartment, whereas the original interpretation relied on a Monocle2/DDRTree trajectory whose root was specified by the user rather than inferred from the data^52,53^. Although *Vcam1* has historically been regarded as absent from normal media and induced only after lesion formation^54,55^ we detected it in a discrete baseline Primed population across all four cohorts. This finding reframes lesional *Vcam1*^+^ medial SMCs as an expansion of a pre-existing compartment rather than a product of de novo *Vcam1* induction.

An adventitial *Ly6a*^+^/*Cd34*^+^ progenitor population (AdvSca1-SM), reported to arise from mature SMCs through Klf4-dependent reprogramming^14^, shares substantial molecular features with disease-expanded Primed SMCs. These include *Cd34* and *Ly6a* expression and a *Lum/Dcn/Pi16/Scara5* fibroblast signature that overlaps our pan-vascular core. However, the retained contractile programme of Primed SMCs is more consistent with a medial than an adventitial location. The two populations may therefore represent related components of a shared progenitor-like SMC lineage whose precise relationship requires spatial resolution.

The strongest functional evidence for a medial *Cd34*^*+*^ SMC progenitor comes from a recent dual-recombinase ablation study that identified a Cd34^+^ smooth-muscle-derived transient progenitor (STPC)^29^. This population generated most neointimal SMCs, and its ablation attenuated hyperplasia, providing independent functional support for autonomous expansion. Although the study concluded that the *Cd34*^*+*^ state was acquired after injury because it was undetectable by immunostaining in uninjured arteries, their single-cell re-clustering identified a discrete *Cd34*^*+*^ subcluster at baseline (1.25% of SMCs) that expanded after injury (12.4% at two weeks). An independent acute-injury dataset similarly detected a Cd34^+^ Primed fraction at baseline (16.1%) that was not induced 3 days after injury (2.7%), despite expansion of the overall compartment^28^.

Moreover, *Dclk1*, the proposed mediator in the ablation study^29^, was enriched in the baseline Primed SMC in the current study across all four cohorts and increased further during disease. Together, these findings suggest that *Cd34* and *Dclk1* are constitutive features of a pre-existing compartment rather than injury-induced acquisitions by contractile SMCs.

The autonomous-expansion architecture also resolves the standing paradox that Sca1-CreERT^2^ tracing minimally labels lesional SMC^12,13^ while scRNA-seq atlases repeatedly identify *Ly6a*/Sca1^+^ populations driving expansion. *Ly6a* is acquired during expansion of the homeostatic Primed pool, not at induction, so prior Sca1-CreERT^2^ before injury would not mark these injury-emergent fraction once compartmental commitment has occurred. The constitutive *S100b*^+^ multipotent population we previously traced with S100b-CreERT^20–22^ likely marks the minor but significant *S100b*^+^ fraction (~7%) of homeostatic Primed SMC, offering a specific but low-coverage genetic handle. Consistent with this interpretation, adenoviral *S100b* knockdown attenuates injury-induced neointima and blocks the formation of a *Ly6a*/Sca-1^+^/α-SMA^+^ intermediate resembling the Primed SMC population identified here^56,57^. By contrast, intersectional *Myh11* tracing gated by the more broadly expressed *Cd34* or *Vcam1* would label a larger proportion of the compartment.

Therapeutically, the phenotypic-switching paradigm has framed SMC modulation as a global transition to be suppressed across the contractile majority^44,45^. The autonomous-expansion model instead focuses attention on a rare, transcriptionally defined Primed compartment whose expansion contributes to lesion growth. Broad anti-proliferative strategies directed at contractile SMCs may therefore affect cells that are not the principal contributors to lesion-associated populations. In contrast, *Cd34* and *Vcam1* define a FACS-isolatable Primed population, enabling targeted functional analysis, perturbation and in vivo screening. Its constitutive matrix and adhesion programmes—including ECM–receptor interactions, AGE–RAGE signalling and proteoglycan biology—also identify potential therapeutic targets, although any intervention would need to preserve their roles in normal vascular homeostasis.

A key limitation is that kNN feeder-source analysis, like trajectory reconstruction more broadly, infers transcriptional proximity to candidate pre-existing states rather than direct clonal ancestry. It therefore cannot formally exclude convergence of a contractile subset on the Primed state^53^. Nevertheless, multicolour clonal-tracing studies have established oligoclonal SMC accumulation from a restricted Myh11^+^ subset^7,9,10^. Together with the repeated identification of the same homeostatic Primed compartment, its dominant self-feeding, its consistent contribution to downstream populations and the reciprocal depletion of contractile SMCs as a source, these findings identify Primed SMCs as the best-supported ancestral compartment currently detectable. Definitive proof will require prospective barcoding, for example by combining an SMC-specific Cre driver with CARLIN^58^ or DARLIN^59^ and somatic mtDNA-based clonal reconstruction in human lesions^60^

Spatially resolved X-inactivation analysis using a BGN 3′-UTR deletion has shown that clonal intimal SMCs correspond to dominant medial clones, supporting a medial origin^61^. The same study identified a predominantly polyclonal lesion with a smaller clonal component, a pattern compatible with autonomous expansion of a pre-existing pool distributed across an extended medial territory. Such expansion could appear more oligoclonal in smaller murine lesions analysed using a limited colour palette. Emerging single-cell mtDNA genotyping has likewise identified spatially restricted and state-biased clonal SMC expansion in the human aorta^62^. However, neither approach has yet resolved the identity of the founder clones. Applying mtDNA-based reconstruction within the Primed-versus-contractile framework therefore represents a critical test in humans.

Additional limitations include the use of a single pooled sample for the carotid dataset and the restriction of the lineage-tracing analyses to males because Myh11-CreERT^2^ is Y-linked. Recovery of the conserved Primed identity in the non-sex-restricted human aortic dataset indicates that the compartment itself is not male-specific. However, potential sex differences in its expansion dynamics will require female-inclusive lineage-tracing models.

In summary, analyses of carotid injury, healthy Myh11-lineage-traced aortic media and two atherosclerosis models converge on a shared vessel-wall architecture. This architecture identifies a rare, constitutively present, progenitor-like Primed SMC compartment as the dominant inferred ancestral source of lesion-associated SMC populations. The findings are most consistent with autonomous expansion and diversification of this pre-existing compartment rather than widespread phenotypic switching of the contractile medial majority. This model reframes the cellular basis of lesion-associated SMC populations, identifies a potential therapeutic target present before disease onset and provides a testable roadmap for future lineage-resolved studies.

## Extended Data

**Extended Data Fig. 3.**
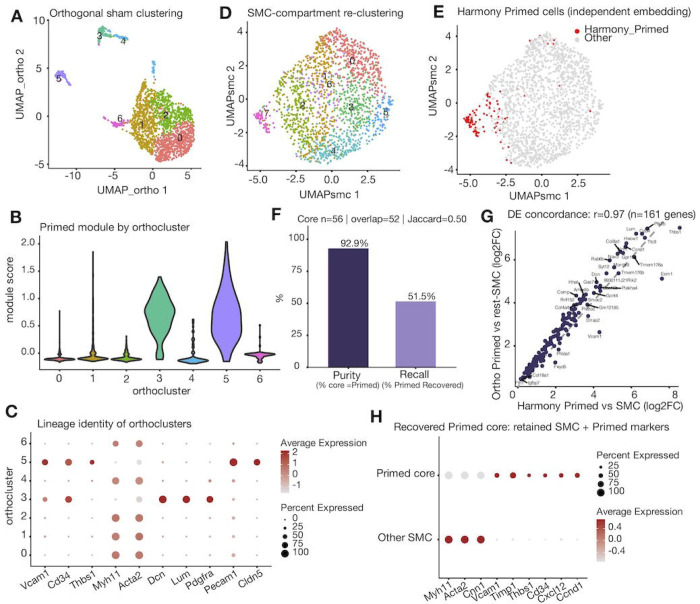
An independent, integration-free re-clustering recovers the Primed SMC pool. (A) Orthogonal re-clustering of sham (Group 1) cells using a separate PCA, graph and UMAP, resolving seven clusters (OrthoC1–7). (B,C) Primed module score (B) and lineage-marker dot plot (C) across orthoclusters: the two module-high whole-sham clusters are adventitial fibroblast (Dcn, Lum, Pdgfra) and endothelial (Pecam1, Cldn5), not SMC — the module cross-reacts with these lineages. (D,E) Re-clustering restricted to the SMC compartment (D), with integrated (Harmony) Primed SMC overlaid in red (E); these cells occupy a coherent region of the independent embedding. (F) A single SMC sub-cluster (the recovered Primed core, n = 56) is 92.9% Harmony Primed and recovers 51.5% of the Primed pool (overlap 52/101; Jaccard 0.50). (G) DE concordance: log_2_FC of the orthogonal Primed-versus-rest-SMC contrast against the integrated Primed-versus-SMC contrast for shared significant genes (Pearson r = 0.97; n = 161). (H) The recovered core retains contractile identity (*Myh11, Acta2, Cnn*1) while expressing Primed markers (*Vcam1, Timp1, Thbs1, Cd34, Cxcl12, Ccnd1*).

**Extended Data Fig. 4.**
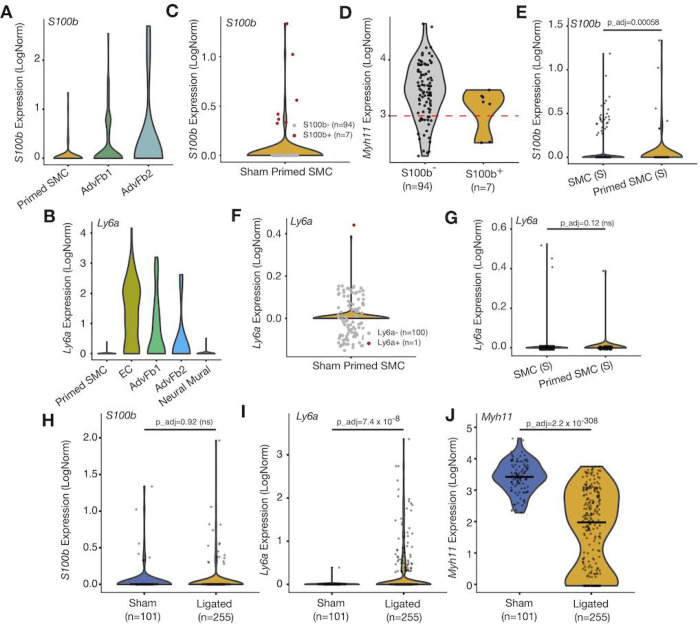
S100b and Ly6a in Primed SMC at homeostasis and after carotid ligation. (A) *S100b* across sham vascular populations — highest in adventitial fibroblast (AdvFb1/2), with only a minority of Primed SMC positive. (B) *Ly6a* — highest in endothelium and adventitial fibroblast, low in Primed SMC and mural cells. (C) *S100b* in sham Primed SMC: S100b^+^ cells (n = 7/101, red) are a minor fraction. (D) *Myh11* in sham S100b^+^ versus S100b^−^ Primed SMC: S100b^+^ cells retain contractile Myh11 within the S100b^−^ range (S100b^+^ median 3.25 vs S100b^−^ 3.47; 5/7 above the Myh11 > 3 gate), confirming SMC identity (n = 7; descriptive). (E) *S100b* is enriched in sham Primed versus contractile SMC (p_adj = 5.8 × 10^−4^). (F) *Ly6a* is absent in sham Primed SMC (n = 1/101). (G) *Ly6a* does not differ between sham Primed and contractile SMC (p_adj = 0.12, n.s.). (H–J) Full Primed SMC cluster, sham (n = 101) versus ligated (n = 255): *S100b* unchanged (p_adj = 0.92; H); Ly6a induced (p_adj = 7.4 × 10^−8^; I); Myh11 reduced (p_adj < 2.2 × 10^−308^; J), the ligated distribution resolving into a Myh11-retaining majority and a Myh11-low fraction. Wilcoxon, BH-corrected; small sham-only subsets (C, D, F) shown descriptively.

**Extended Data Fig. 5.**
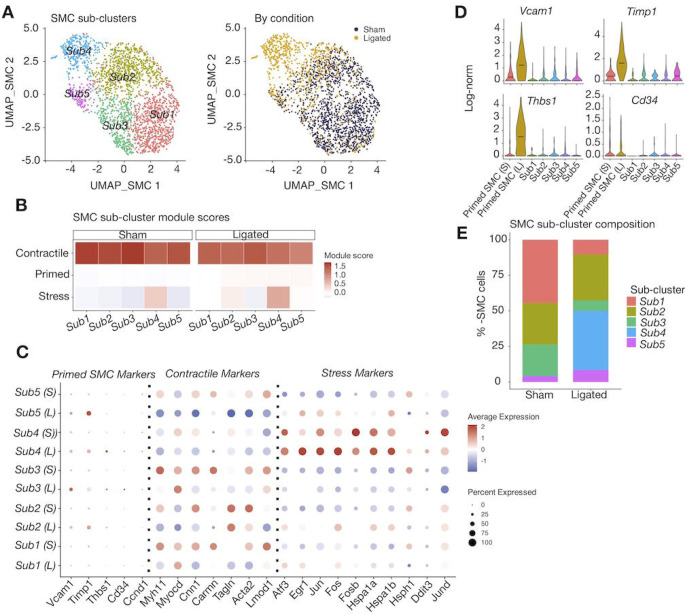
Sub-clustering of the contractile SMC compartment resolves quiescent sub-states selectively depleted by injury. The pooled contractile SMC cluster (sham + ligated; n = 2,295) was re-clustered (Harmony over condition, resolution 0.5), resolving five sub-clusters (Sub1–Sub5). (A) UMAP by sub-cluster (left) and condition (right). (B) Mean contractile, Primed and stress module scores per sub-cluster by condition: contractile identity is retained throughout, the Primed module stays near-baseline (confirming these are contractile, not Primed, SMC), and stress is elevated in Sub4. (C) Dot plot of Primed, contractile and stress markers across sub-clusters by condition (S, sham; L, ligated; dot size, % expressing; colour, scaled mean). (D) Violins of *Vcam1, Timp1, Thbs1, Cd34* in sham/ligated Primed SMC and each sub-cluster: Primed markers are enriched in Primed SMC and minimal across sub-states (bars, medians). (E) Sub-cluster composition by condition: quiescent Sub1–Sub3 are depleted after ligation (1,468 → 381; 3.9×), while stress sub-state Sub4 is almost entirely ligation-emergent (98.4% ligated).

**Extended Data Fig. 6.**
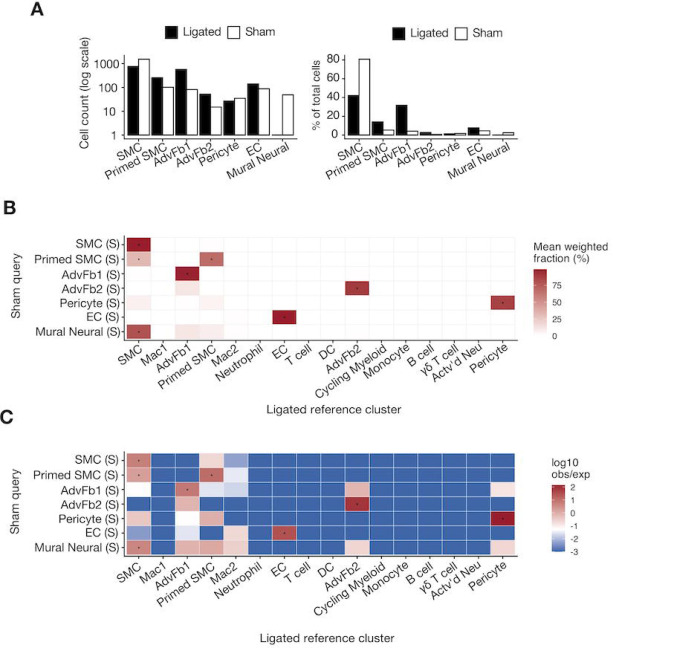
Reciprocal (Sham→Ligated) kNN mapping shows each resident population persists as its own ligated counterpart. (A) Mesenchymal/vascular composition in sham and ligated carotid: absolute counts (left, log scale) and % of captured cells (right); the Primed pool expands as contractile SMC contract. (B,C) Reciprocal kNN mapping, each sham population (rows, query) to its nearest ligated neighbours (columns, reference; k = 15, 20 Harmony dimensions): mean weighted neighbour fraction (B) and enrichment over background (C, log_10_ obs/exp). Asterisks, mappings significant after Bonferroni correction (permutation test, N = 500); at this depth the corrected floor is P ≈ 0.032 (≈0.0020 × 16 clusters), so all significant mappings read as a single asterisk (*P < 0.05). Each sham population maps to its own ligated counterpart along the diagonal — sham Primed SMC (n = 101) → ligated Primed SMC (~11×); sham contractile SMC (n = 1,534) → ligated contractile SMC (~5.5×); AdvFb1/AdvFb2/pericyte/endothelium to like — with the high enrichments for the rarest queries (AdvFb2, n = 14; pericyte, n = 34) amplified by low background frequency. Sham contractile SMC are depleted as a source of ligated Primed SMC (0.22×), mirroring Fig. 4; the modest proximity of sham Primed to ligated contractile SMC (~1.8×) reflects the poised contractile programme retained by Primed SMC, not directional flux. Mural neural cells, absent from the ligated capture, map to ligated contractile SMC by default.

**Extended Data Fig. 7.**
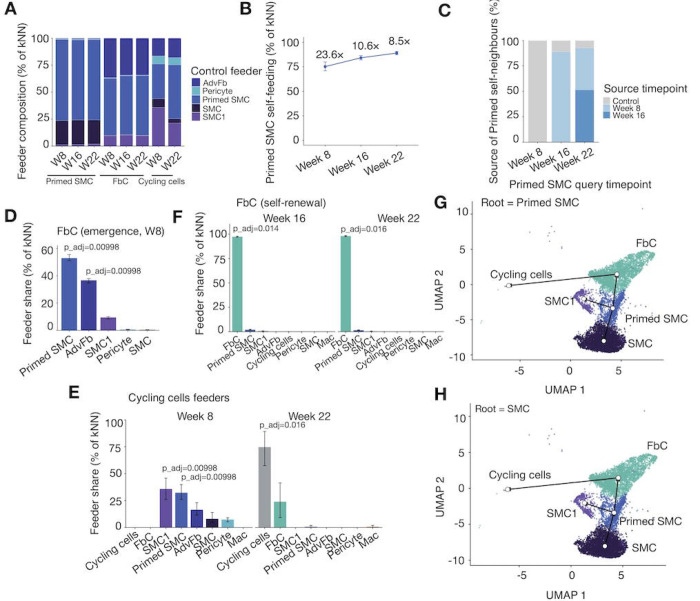
Autonomous expansion and self-renewal of the Primed SMC pool in the ApoE^−^/^−^ model. Forward kNN feeder-source and dual-rooted Slingshot analysis of lineage-positive (ZsGreen1-WPRE > 3.17) ApoE^−^/^−^ cells (Control, Weeks 8/16/22; k = 15, 20 Harmony dimensions). Bars/points, feeder share; error bars, bootstrap 95% CI (50 iterations); p_adj, 500-permutation test, Bonferroni-corrected. The floor scales with cluster number — 0.00998 (five, Week 8), ≈0.014 (seven, Week 16), ≈0.016 (eight, Week 22) — so the strongest later associations read * (p_adj < 0.05) not **. (A) Feeder composition of each query (Primed SMC, FbC, Cycling cells) by source cluster: Primed queries are self-dominated; FbC draw from Primed SMC and AdvFb; Cycling cells from SMC1, Primed SMC and AdvFb (the SMC1 contribution is a cell-cycle effect; see E). (B) Primed SMC self-feeding >75% at every timepoint (fold-enrichment annotated). (C) Temporal source of Primed self-neighbours: later Primed SMC are seeded by earlier Primed SMC, not contemporaneous recruitment. (D) Emerging FbC (Week 8) fed chiefly by Primed SMC and AdvFb (each p_adj = 0.00998), with minor non-significant SMC1 and negligible SMC/pericyte input. (E) Cycling cells — Week 8: SMC1 and Primed SMC (each p_adj = 0.00998); Week 22: predominantly self-renewing with an FbC contribution (p_adj = 0.016). Cell-cycle-phase regression resolves the Week-8 SMC1 proximity onto AdvFb and Primed SMC and markedly reduces Week-22 self-renewal, identifying both as cell-cycle-embedding effects (Supplementary Fig. 5A,B,D); the Week-22 population is small (n = 10) and k-sensitive. (F) FbC at Weeks 16 and 22 maintained almost entirely by self-neighbours (p_adj = 0.014, 0.016). (G,H) Dual-rooted Slingshot over the ApoE SMC-fate compartment (SMC, SMC1, Primed SMC, FbC, Cycling cells; 10 Harmony dimensions): rooting at Primed SMC (G) places both contractile clusters terminal; rooting at SMC (H) forces Primed SMC to an intermediate. Topology only; direction is adjudicated by the feeder analysis (B–F). FbC, fibromyocyte–fibrochondrocyte (C2+C7); SMC1, minor activated-contractile subset; AdvFb, adventitial fibroblast.

**Extended Data Fig. 8.**
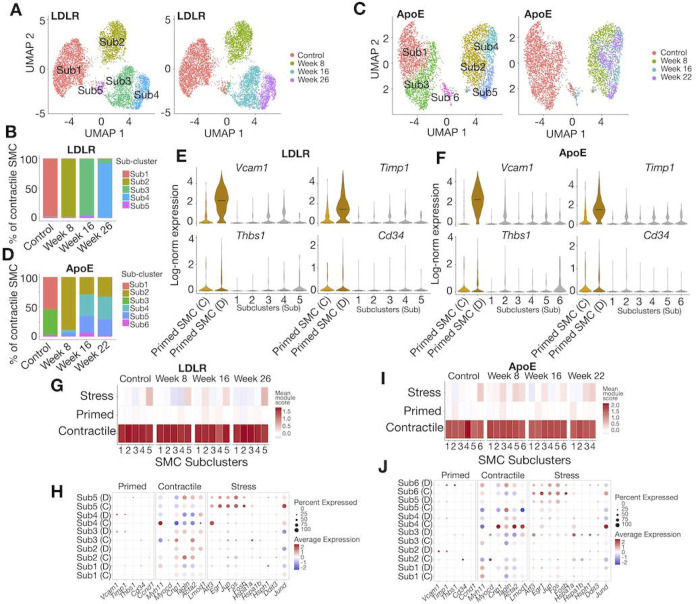
Disease-associated contractile SMC subclusters do not acquire the Primed programme in the LDLR and ApoE cohorts. (A) LDLR contractile SMC subclusters by subcluster (Sub1–5, left) and timepoint (Control, Weeks 8/16/26, right). (B) Subcluster composition across LDLR timepoints (% of contractile SMC). (C,D) As A,B for ApoE (Sub1–6; Control, Weeks 8/16/22). (E) Violins of Primed markers (Vcam1, Timp1, Thbs1, Cd34) in control (C) and disease (D) Primed SMC versus each LDLR subcluster — expression restricted to Primed SMC. (F) As E for ApoE. (G) Mean stress, Primed and contractile module scores across LDLR subclusters per timepoint: contractile dominates, Primed stays near zero. (H) Dot plot of Primed, contractile and stress markers across LDLR subclusters, control (C) and disease (D). (I,J) As G,H for ApoE.

**Extended Data Fig. 9.**
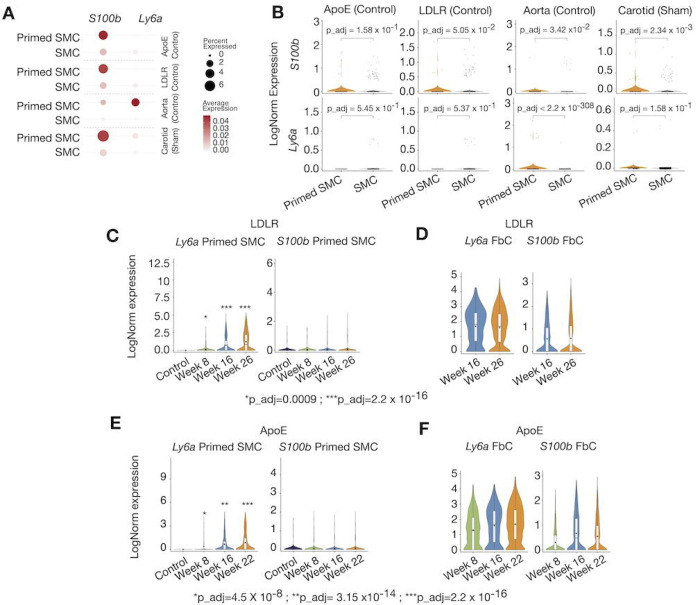
S100b and Ly6a in Primed SMC at baseline and across disease progression. (A) Dot plot of S100b and Ly6a in Primed versus contractile SMC at baseline across four cohorts (ApoE/LDLR/Aorta control, Carotid sham; dot size, % expressing; colour, mean expression). (B) Violins of S100b (top) and Ly6a (bottom) in Primed versus contractile SMC per baseline cohort (pairwise p_adj indicated). (C) Ly6a and S100b in LDLR Primed SMC (Control, Weeks 8/16/26). (D) Ly6a and S100b in LDLR FbC (Weeks 16, 26). (E,F) As C,D for ApoE (Primed SMC: Control, Weeks 8/16/22; FbC: Weeks 8/16/22). Asterisks (C, E), pairwise p_adj versus Control (Wilcoxon, BH-corrected); D and F show FbC across available timepoints (no Control, FbC absent at baseline).

**Extended Data Fig. 10.**
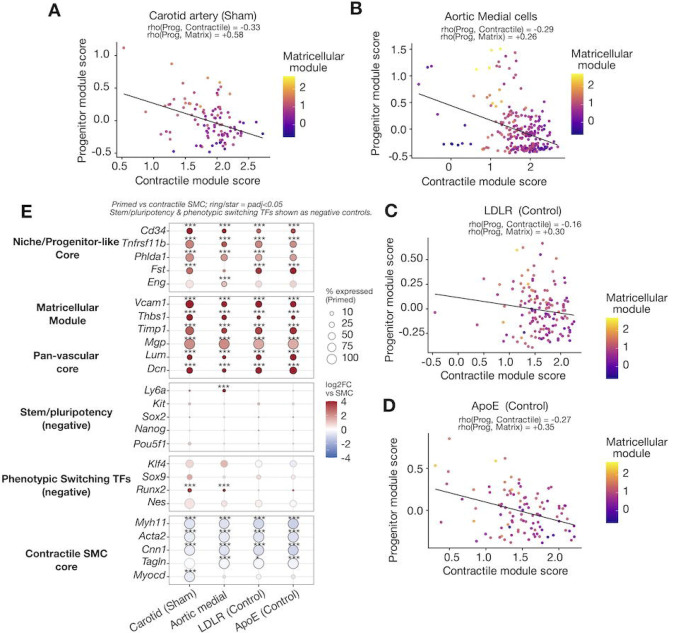
The Primed programme co-varies with the matricellular but not the contractile or phenotypic-switching programmes. (A–D) Per-cell scatter of progenitor versus contractile module score, coloured by matricellular score, with Spearman ρ, for Carotid (Sham; A), Aortic medial (B), LDLR Control (C) and ApoE Control (D): the progenitor module anticorrelates with contractile and correlates with matricellular, and the matricellular module is inversely related to contractile (ρ = −0.32 to −0.45). (E) Dot plot of Primed-versus-contractile log_2_FC across six modules — progenitor-like, matricellular, pan-vascular core, stem/pluripotency (negative control), phenotypic-switching TFs (negative control), contractile core — in four baseline cohorts (dot size, % expressing in Primed; colour, log_2_FC; ring/star, p_adj < 0.05). Primed cells up-regulate progenitor-like, matricellular and pan-vascular genes and down-regulate the contractile core. Stem/pluripotency markers show no enrichment (Ly6a significant only in the aorta); phenotypic-switching TFs Klf4, Sox9 and Nes are not enriched in any cohort; Runx2 is enriched only in carotid and aorta.

## Supplementary Material

Supplement 1 Supplementary Fig. 1. Transcript-level lineage definition excludes phagocytic myeloid false-positives across both atherosclerosis cohorts.
lineage is marked by the Myh11-CreERT2–driven ZsGreen reporter, so ZsGreen protein in a non-SMC cell is SMC-lineage–derived by definition. Each cell was cross-classified by FACS sort status and transcript-level reporter detection (ZsGreen1-WPRE > 3.17) into True Lineage (FACS+/transcript+), Discordant (FACS+/transcript−), Gate-missed (FACS−/transcript+) and Negative (FACS−/transcript−). Group sizes (LDLR/ApoE): 11,657/10,699; 3,667/2,599; 299/289; 11,050/10,455. (A–C) LDLR; (D–F) ApoE. (A,D) Discordant cells per Harmony cluster, coloured by provisional identity (SMC, immune, fibroblast, EC; argmax of lineage-marker means): they localise predominantly to immune clusters (LDLR 54.4%, ApoE 53.1%) and only marginally to SMC (8.8%, 4.5%). (B,E) Marker dot plot across the four groups (SMC, myeloid/immune, efferocytosis genes and the reporter transcript; dot size, % expressing; colour, scaled mean). True Lineage cells co-express the SMC programme and high reporter transcript, whereas Discordant cells express the myeloid/immune programme with low reporter transcript despite FACS positivity (median ZsGreen1-WPRE — True Lineage 4.38/4.46, Discordant 1.45/1.29, Gate-missed 3.39/3.42, Negative 0.79/0.00). Per cell, 66.0%/69.3% of Discordant cells are myeloid-marker^+^ versus 27.5%/25.1% SMC-marker^+^/immune-marker^−^ (genuine dropout; n = 1,007/653); mean module scores immune +0.70/+0.73, SMC −0.52/−0.58, efferocytosis −0.10/−0.04 — i.e. myeloid cells carrying ingested reporter protein, not an active efferocytosis signature. (C,F) Harmony UMAP with Discordant cells (red) mapping predominantly to myeloid territory.

Supplement 2 Supplementary Fig. 2. Expression-based definition of the ZsGreen1-WPRE lineage threshold (LDLR^−^/^−^ reference cohort).
Lineage-positive cells were defined by ZsGreen1-WPRE log-normalised expression > 3.17, set by maximising Youden's J against the FACS sort gate (n = 26,673; AUC = 0.90, sensitivity = 0.76, specificity = 0.97) and applied globally to both cohorts and all timepoints. (A) Reporter expression in the two FACS fractions (dashed line, > 3.17); the sort-positive fraction contains a substantial sub-threshold population (imperfect protein gating). (B) The same by transcript-defined status at the > 3.17 cutoff. (C) Kernel density across all cells; the distribution is bimodal, with the threshold separating the modes. (D) FACS gate versus transcript threshold: concordant 11,657 (positive) and 11,050 (negative); discordant 3,667 (sort-positive, transcript-negative; under-captured) and 299 (sort-negative, transcript-positive; recovered recombined cells). (E) UMAP of all LDLR^−^/^−^ cells coloured by reporter expression. (F) The same, lineage-positive cells (> 3.17) coloured by expression; negative cells grey.

Supplement 3 Supplementary Fig. 3. Ligation-associated reductions in library complexity are confined to contractile SMC.
(A) Whole-atlas QC: UMI count and genes detected, Sham versus Ligated; ligation lowers median UMIs by 45% and genes by 30% (both p < 2.2 × 10^−308^). (B) Per-cluster signed –log_10_p (Ligated versus Sham) for nCount and nFeature: the reduction is driven almost entirely by contractile SMC, with other lineages non-significant or modest (purple, lower in Ligated; amber, higher). (C) Focused QC: contractile SMC show reduced UMIs (–27%) and genes (–14%; both p < 2.2 × 10^−308^), whereas Primed SMC are unchanged (UMI +8%, p = 1.8 × 10^−1^; genes +4%, p = 2.2 × 10^−1^) (box, median/IQR; red point, mean). Primed SMC expansion is therefore not attributable to library quality or ambient RNA

Supplement 4 Supplementary Fig. 4. Lineage-tracing recovery, gate validation and labelling reproducibility across the LDLR and ApoE cohorts.
Colour denotes model: Shared (common baseline Control), LDLR (green), ApoE (blue). (A) Total cells recovered per timepoint per cohort. (B) Cluster-resolved counts (contractile SMC, Primed SMC, FbC) across timepoints, faceted by cohort: contractile SMC decline as Primed SMC and FbC accumulate. (C) Lineage^+^ (ZsGreen^+^) cells per timepoint and cohort. (D) Cluster composition as a fraction of lineage^+^ cells: the SMC fraction falls, the FbC fraction rises. (E) Lineage^+^ fraction, stable (~40–48%) across cohorts and timepoints. (F) Expression-gate validation against FACS: FACS-negative controls recover essentially no ZsGreen^+^ cells, while FACS-positive controls and disease samples do, confirming concordance with the FACS ground truth.

Supplement 5 Supplementary Fig. 5. Cycling cells are the proliferative arm of the Primed→FbC axis; their apparent SMC1 origin and late self-renewal are cell-cycle artefacts, and feeder calls are robust to neighbourhood size.
(A) Control feeder share of Week-8 ApoE^−^/^−^ cycling cells (k = 15, 20 Harmony dimensions; n = 19) before versus after S/G2M-phase regression and re-embedding (p_adj < 0.01; 500 **p**ermutations, Bonferroni): regression abolishes SMC1 (11.1× → **2**.1×, n.s.) and resolves origin onto AdvFb (→ **7.7**×) and Primed SMC (→ 4.0×). (B) Fate of the 32 cycling cells after cell-cycle-corrected re-clustering, by dominant non-cycling identity: predominantly FbC (69%) and Primed SMC (9%). (C,D) Feeder share versus neighbourhood size (k = 5–50) at emergence (solid) and late (dashed) timepoints (dotted line, k = 15). (C) LDLR^−^/^−^ (Weeks 16, 26), original embedding: emergence fed by Primed SMC (~59% for k ≥ 15); the Week-26 self-renewal estimate is strongly k-dependent (~94% → ~60% → ~19% at k = 5/15/50). (D) ApoE^−^/^−^ (Weeks 8, 22), cell-cycle-corrected: Week-8 origin is AdvFb + Primed SMC across all k with SMC1 minimal; by Week 22 cells map mainly to FbC with only minor self-feeding. Late-timepoint self-renewal rests on small populations (LDLR n = 22; ApoE n = 10) and indicates tendencies, not fixed values; the Primed and FbC calls carrying the main conclusions are k-independent.

Supplement 6 Supplementary Fig. 6. Integration-free cross-cohort corroboration of the Primed SMC identity.
Cohort fold-change similarity (CFS) scores transcriptome-wide concordance between per-cluster fold-change signatures computed independently within each cohorťs uncorrected RNA assay, without integration or batch correction (values symmetric). Baseline/uninjured cells only (sham carotid; control LDLR/ApoE; healthy aortic medial), lineage-gated where applicable (ZsGreen > 3.17 for LDLR/ApoE; Confetti-sorted medial), restricted to the shared mesenchymal roster. Baseline Primed SMC: carotid n = 101, LDLR n = 141, ApoE n = 100, aortic medial n = 240 (SMC identity lineage-confirmed in three cohorts; transcriptionally defined in carotid, 79/101 Myh11 > 3). (A) Cross-cohort Primed↔Primed CFS matrix; boxed = reciprocal-best. Five of six pairs positive, carotid↔aortic media (−1.1) the exception. (B) Each source cohorťs Primed SMC (rows) scored against partner Primed SMC (amber) and partner adventitial fibroblast (green) clusters; FACS-sorted aortic medial source shaded. (C) As (B) under cell-number-balanced downsampling (120 cells/type; robustness control).

Supplement 7 Supplementary Fig 7. Two orthogonal trajectory methods independently identify the Primed SMC as the bridge to the disease-state compartment.
Five mural roles (contractile SMC, Primed SMC, fibromyocyte, fibrochondrocyte, cycling) in the LDLR (DS3) and ApoE (DS5) cohorts, on a shared layout. **(A)** Connectivity, no time axis. PAGA-style observed/expected kNN connectivity (abstracted graph, top; symmetric matrix, bottom; shared key 0–0.36). The Primed SMC is the hub linking the contractile pole to the disease states (LDLR Primed–fibrochondrocyte 0.36, Primed–fibromyocyte 0.31; ApoE Primed–fibromyocyte 0.24 → fibromyocyte–fibrochondrocyte 0.36). The contractile SMC connects only to the Primed SMC (0.19 / 0.18) and is otherwise inert toward the disease compartment (≤0.08; contractile–fibromyocyte ≈0). (B) Directed flow across time. Temporal-flow enrichment: per adjacent timepoint pair (Control → Wk8 → Wk16 → Wk26 [LDLR] / Wk22 [ApoE]), each later-stage cell's 30 nearest neighbours among earlier-stage cells were scored by role over 20 dimensions; the matrix gives observed/expected enrichment of each source role (earlier stage; rows) for each target role (later stage; columns), diverging key 1/16–16 (1 = expected). Every disease state is self-sustaining once it emerges (diagonal: cycling 64.6 / 39.1, fibrochondrocyte 12.5 / 9.3, fibromyocyte 4.0 / 2.6), and among the baseline states only the Primed SMC self-renews (5.0 / 5.9); the contractile SMC feeds no disease target (≤0.11 in both cohorts).

Supplement 8

Supplement 9

Supplement 10
