# Supplementary material for: A rare pre-existing progenitor-like Primed SMC compartment is the dominant inferred source of SMC-derived cellularity in vascular injury and atherosclerosis
Source: bioRxiv. 2026 Jul 24:2026.06.28.735042. Preprint. [Version 6] doi: 10.64898/2026.06.28.735042 (PMC13420442; doi:10.64898/2026.06.28.735042)
Supplement: Supplementary file 1 [file media-1.pdf]

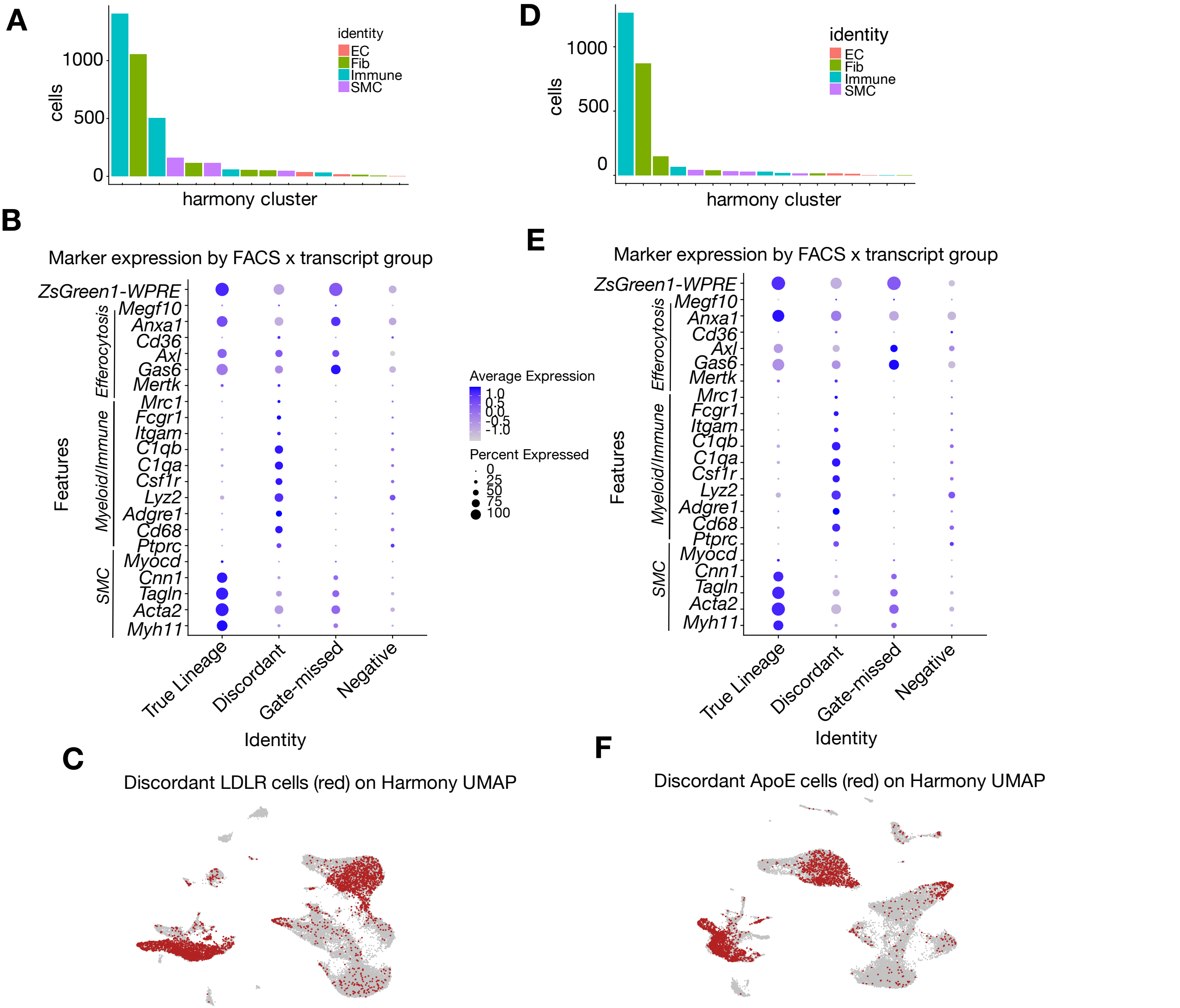
A
identity
EC
Fib
Immune
SMC
1000
500
0
cells
harmony cluster
B
Marker expression by FACS x transcript group
ZsGreen1-WPRE
Megf10
Anxa1
Cd36
Axl
Gas6
Mertk
Mrc1
Fcgr1
Itgam
C1qb
C1qa
Csf1r
Lyz2
Adgre1
Cd68
Ptprc
Myocd
Cnn1
Tagln
Acta2
Myh11
Efferocytosis
Myeloid/Immune
SMC
Features
True Lineage
Discordant
Gate-missed
Negative
Identity
Average Expression
1.0
0.5
0.0
-0.5
-1.0
Percent Expressed
0
25
50
75
100
C
Discordant LDLR cells (red) on Harmony UMAP
D
identity
EC
Fib
Immune
SMC
1000
500
0
cells
harmony cluster
E
Marker expression by FACS x transcript group
ZsGreen1-WPRE
Megf10
Anxa1
Cd36
Axl
Gas6
Mertk
Mrc1
Fcgr1
Itgam
C1qb
C1qa
Csf1r
Lyz2
Adgre1
Cd68
Ptprc
Myocd
Cnn1
Tagln
Acta2
Myh11
Efferocytosis
Myeloid/Immune
SMC
Features
True Lineage
Discordant
Gate-missed
Negative
Identity
F
Discordant ApoE cells (red) on Harmony UMAP
