# Supplementary material for: A rare pre-existing progenitor-like Primed SMC compartment is the dominant inferred source of SMC-derived cellularity in vascular injury and atherosclerosis
Source: bioRxiv. 2026 Jul 24:2026.06.28.735042. Preprint. [Version 6] doi: 10.64898/2026.06.28.735042 (PMC13420442; doi:10.64898/2026.06.28.735042)
Supplement: Supplementary file 2 [file media-2.pdf]

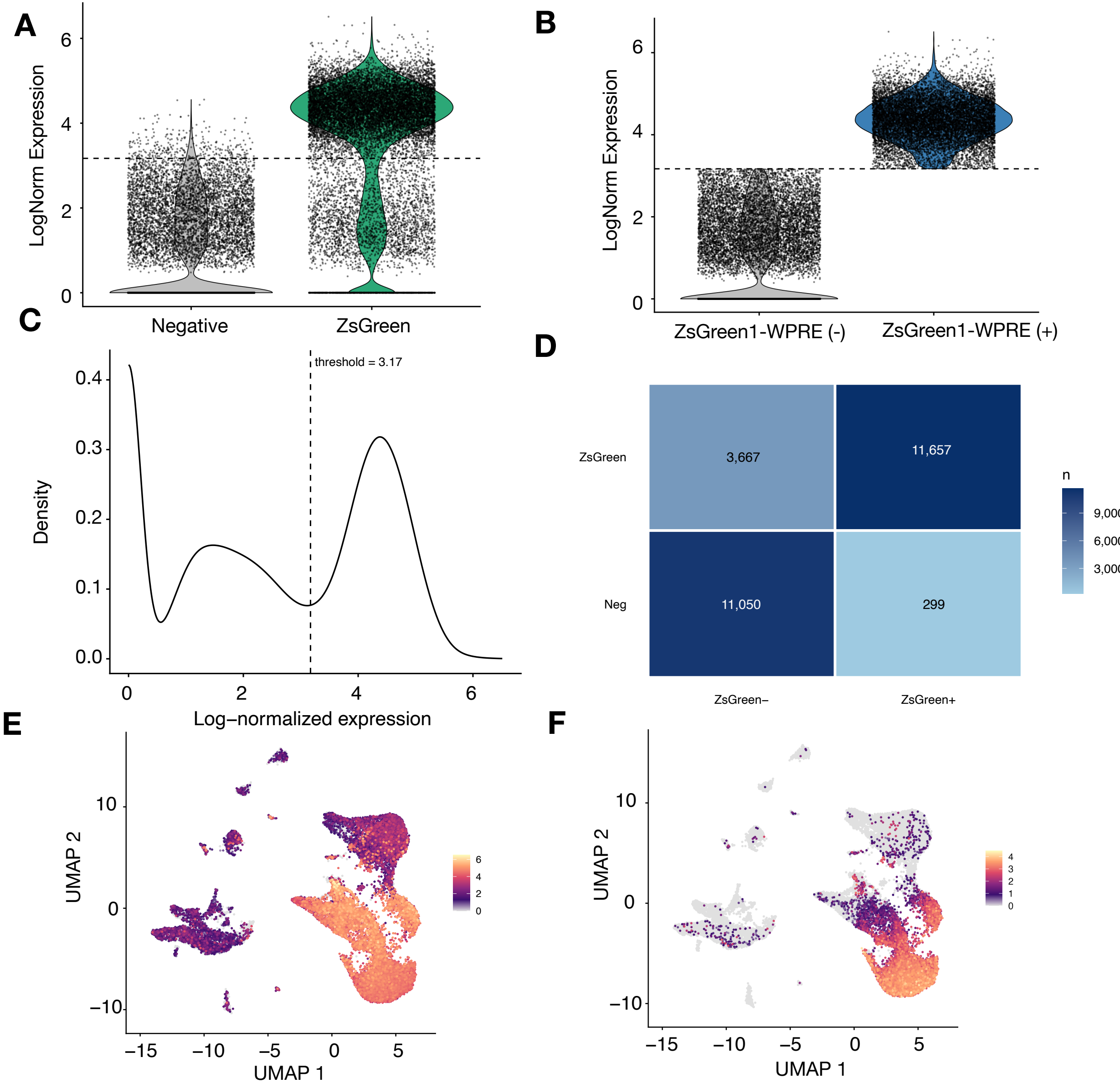

A
LogNorm Expression
Negative
ZsGreen
B
LogNorm Expression
ZsGreen1-WPRE (-)
ZsGreen1-WPRE (+)
C
threshold = 3.17
Density
Log−normalized expression
D
ZsGreen
Neg
3,667
11,657
11,050
299
ZsGreen−
ZsGreen+
n
9,000
6,000
3,000
E
UMAP 2
UMAP 1
F
UMAP 2
UMAP 1
