# Supplementary material for: A rare pre-existing progenitor-like Primed SMC compartment is the dominant inferred source of SMC-derived cellularity in vascular injury and atherosclerosis
Source: bioRxiv. 2026 Jul 24:2026.06.28.735042. Preprint. [Version 6] doi: 10.64898/2026.06.28.735042 (PMC13420442; doi:10.64898/2026.06.28.735042)
Supplement: Supplementary file 3 [file media-3.pdf]

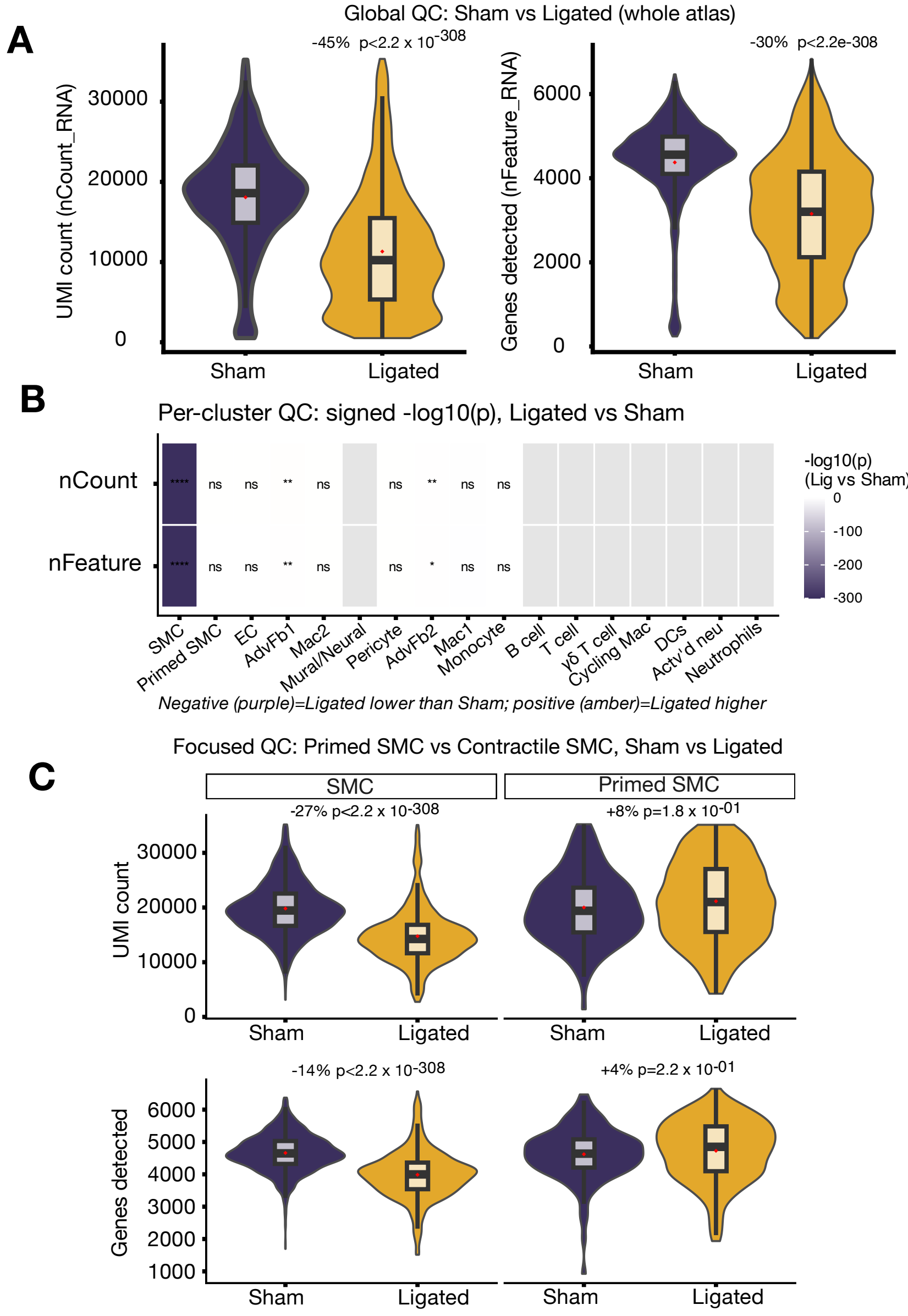
A
Global QC: Sham vs Ligated (whole atlas)
-45% p<2.2 x 10-308
UMI count (nCount_RNA)
30000
20000
10000
0
Sham
Ligated
-30% p<2.2e-308
Genes detected (nFeature_RNA)
6000
4000
2000
0
Sham
Ligated
B
Per-cluster QC: signed -log10(p), Ligated vs Sham
nCount
nFeature
**** ns ns ** ns ns ** ns ns
**** ns ns ** ns ns * ns ns
SMC
Primed SMC
EC
AdvFb1
Mac2
Mural/Neural
Pericyte
AdvFb2
Mac1
Monocyte
B cell
T cell
γδ T cell
Cycling Mac
DCs
Actv'd neu
Neutrophils
-log10(p)
(Lig vs Sham)
0
-100
-200
-300
Negative (purple)=Ligated lower than Sham; positive (amber)=Ligated higher
Focused QC: Primed SMC vs Contractile SMC, Sham vs Ligated
C
SMC
Primed SMC
-27% p<2.2 x 10-308
+8% p=1.8 x 10-01
UMI count
30000
20000
10000
0
Sham
Ligated
Sham
Ligated
-14% p<2.2 x 10-308
+4% p=2.2 x 10-01
Genes detected
6000
5000
4000
3000
2000
1000
Sham
Ligated
Sham
Ligated
