# Supplementary material for: A rare pre-existing progenitor-like Primed SMC compartment is the dominant inferred source of SMC-derived cellularity in vascular injury and atherosclerosis
Source: bioRxiv. 2026 Jul 24:2026.06.28.735042. Preprint. [Version 6] doi: 10.64898/2026.06.28.735042 (PMC13420442; doi:10.64898/2026.06.28.735042)
Supplement: Supplementary file 4 [file media-4.pdf]

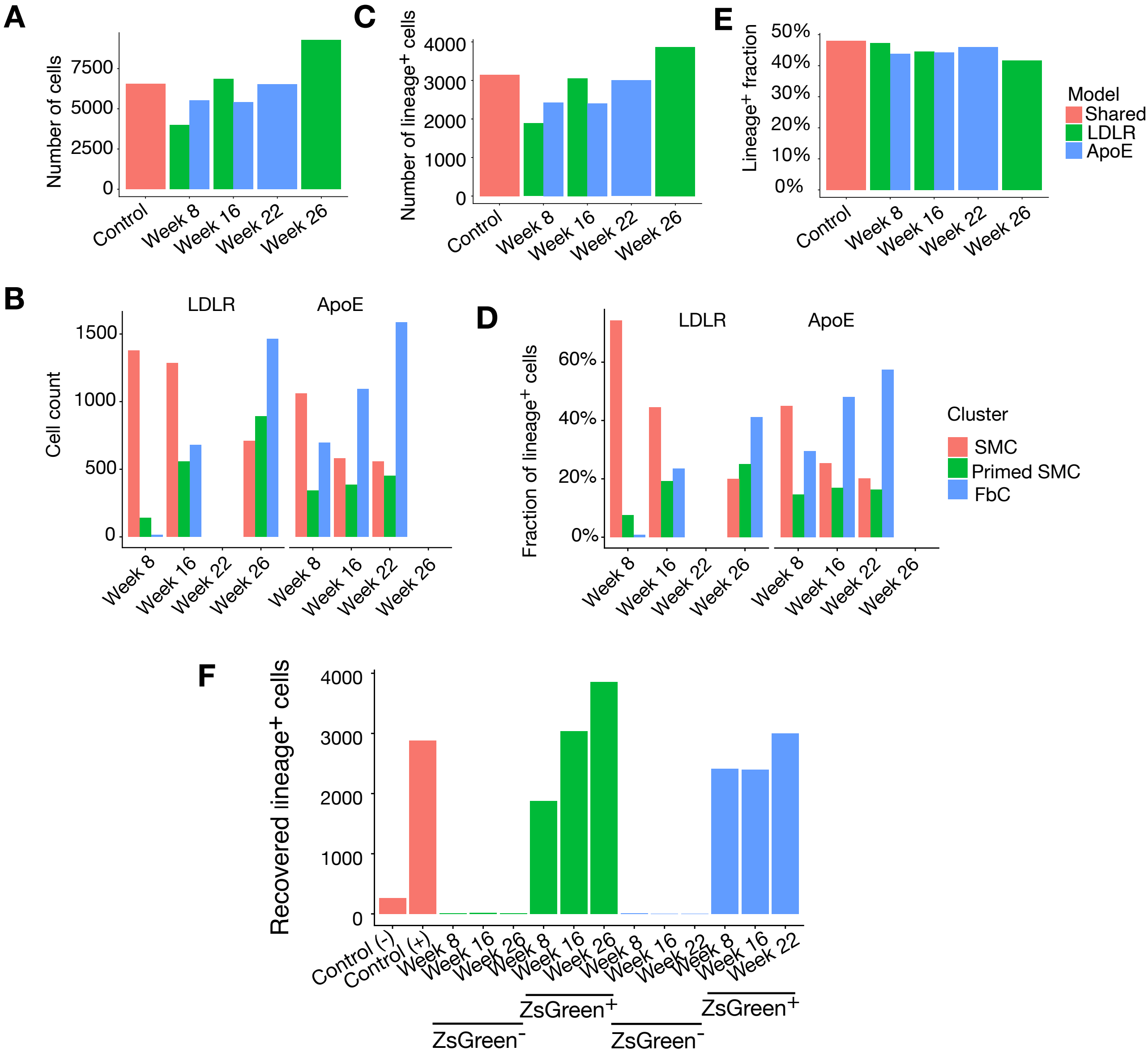
A
Number of cells
7500
5000
2500
0
Control
Week 8
Week 16
Week 22
Week 26
C
Number of lineage+ cells
4000
3000
2000
1000
0
E
Lineage+ fraction
50%
40%
30%
20%
10%
0%
Model
Shared
LDLR
ApoE
B
LDLR
ApoE
Cell count
1500
1000
500
D
Fraction of lineage+ cells
60%
Cluster
SMC
Primed SMC
FbC
F
Recovered lineage+ cells
Control (-)
Control (+)
ZsGreen+
ZsGreen-
