# Supplementary material for: A rare pre-existing progenitor-like Primed SMC compartment is the dominant inferred source of SMC-derived cellularity in vascular injury and atherosclerosis
Source: bioRxiv. 2026 Jul 24:2026.06.28.735042. Preprint. [Version 6] doi: 10.64898/2026.06.28.735042 (PMC13420442; doi:10.64898/2026.06.28.735042)
Supplement: Supplementary file 5 [file media-5.pdf]

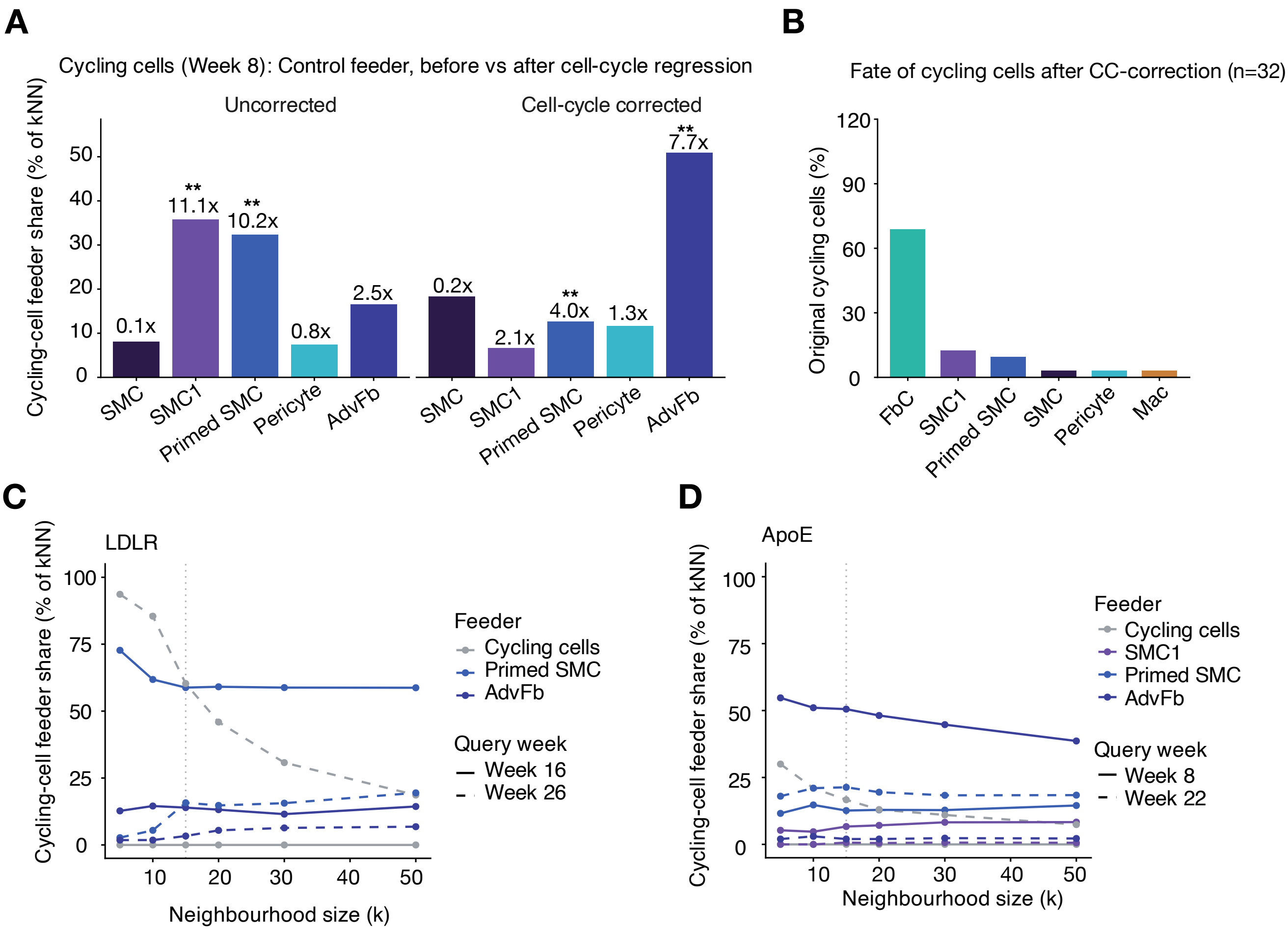

A
Cycling cells (Week 8): Control feeder, before vs after cell-cycle regression
Uncorrected
Cell-cycle corrected
Cycling-cell feeder share (% of kNN)
0.1x
**
11.1x
**
10.2x
0.8x
2.5x
0.2x
2.1x
**
4.0x
1.3x
**
7.7x
SMC
SMC1
Primed SMC
Pericyte
AdvFb
B
Fate of cycling cells after CC-correction (n=32)
Original cycling cells (%)
FbC
SMC1
Primed SMC
SMC
Pericyte
Mac
C
LDLR
Cycling-cell feeder share (% of kNN)
Neighbourhood size (k)
Feeder
Cycling cells
Primed SMC
AdvFb
Query week
Week 16
Week 26
D
ApoE
Cycling-cell feeder share (% of kNN)
Neighbourhood size (k)
Feeder
Cycling cells
SMC1
Primed SMC
AdvFb
Query week
Week 8
Week 22
