# Supplementary material for: A rare pre-existing progenitor-like Primed SMC compartment is the dominant inferred source of SMC-derived cellularity in vascular injury and atherosclerosis
Source: bioRxiv. 2026 Jul 24:2026.06.28.735042. Preprint. [Version 6] doi: 10.64898/2026.06.28.735042 (PMC13420442; doi:10.64898/2026.06.28.735042)
Supplement: Supplementary file 6 [file media-6.pdf]

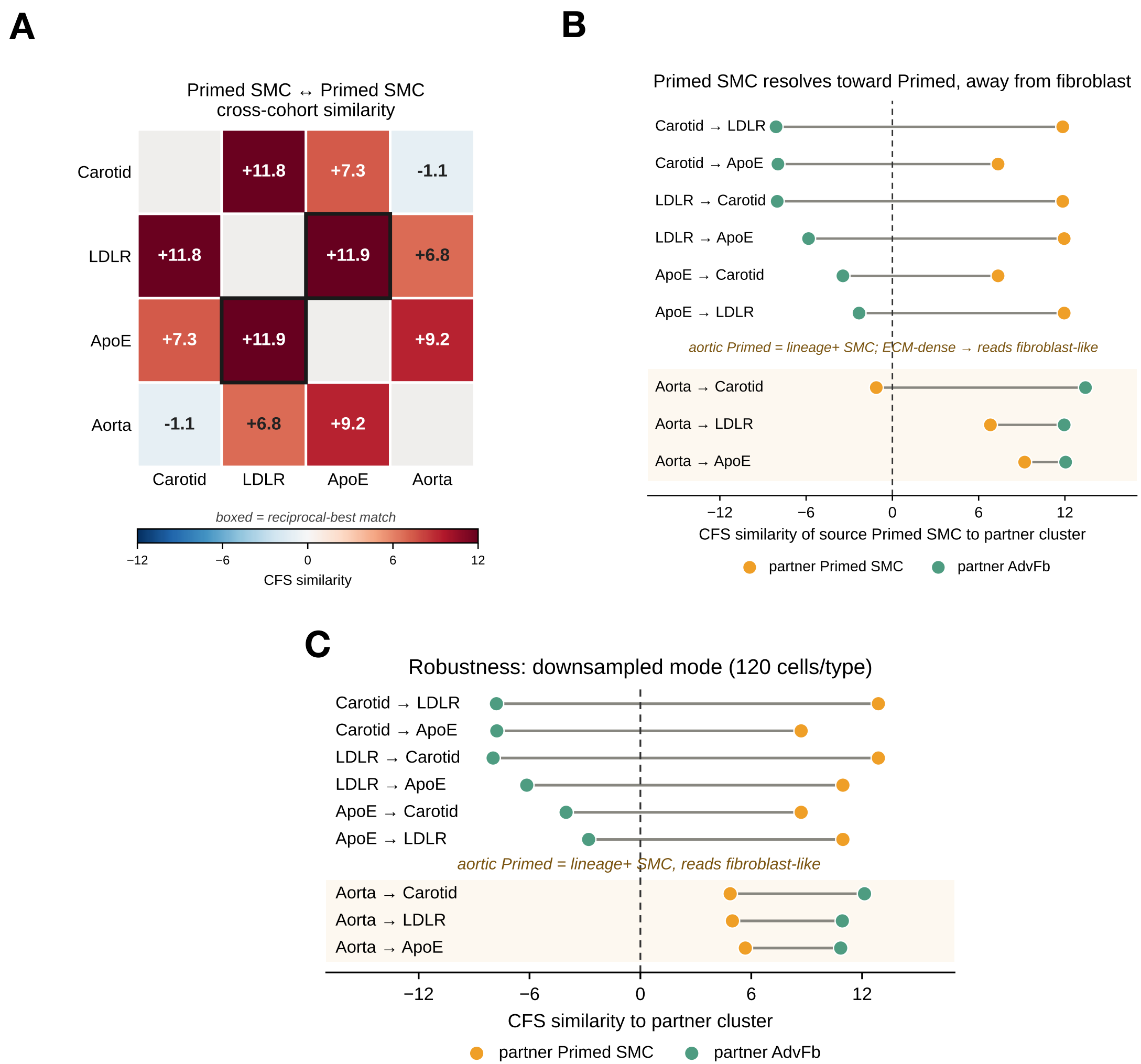
A
Primed SMC ↔ Primed SMC
cross-cohort similarity
Carotid
LDLR
ApoE
Aorta
+11.8
+7.3
-1.1
+11.8
+11.9
+6.8
+7.3
+11.9
+9.2
-1.1
+6.8
+9.2
Carotid
LDLR
ApoE
Aorta
boxed = reciprocal-best match
−12
−6
0
6
12
CFS similarity
B
Primed SMC resolves toward Primed, away from fibroblast
Carotid → LDLR
Carotid → ApoE
LDLR → Carotid
LDLR → ApoE
ApoE → Carotid
ApoE → LDLR
aortic Primed = lineage+ SMC; ECM-dense → reads fibroblast-like
Aorta → Carotid
Aorta → LDLR
Aorta → ApoE
−12
−6
0
6
12
CFS similarity of source Primed SMC to partner cluster
partner Primed SMC
partner AdvFb
C
Robustness: downsampled mode (120 cells/type)
Carotid → LDLR
Carotid → ApoE
LDLR → Carotid
LDLR → ApoE
ApoE → Carotid
ApoE → LDLR
aortic Primed = lineage+ SMC, reads fibroblast-like
Aorta → Carotid
Aorta → LDLR
Aorta → ApoE
−12
−6
0
6
12
CFS similarity to partner cluster
partner Primed SMC
partner AdvFb
