# Supplementary material for: A rare pre-existing progenitor-like Primed SMC compartment is the dominant inferred source of SMC-derived cellularity in vascular injury and atherosclerosis
Source: bioRxiv. 2026 Jul 24:2026.06.28.735042. Preprint. [Version 6] doi: 10.64898/2026.06.28.735042 (PMC13420442; doi:10.64898/2026.06.28.735042)
Supplement: Supplementary file 7 [file media-7.pdf]

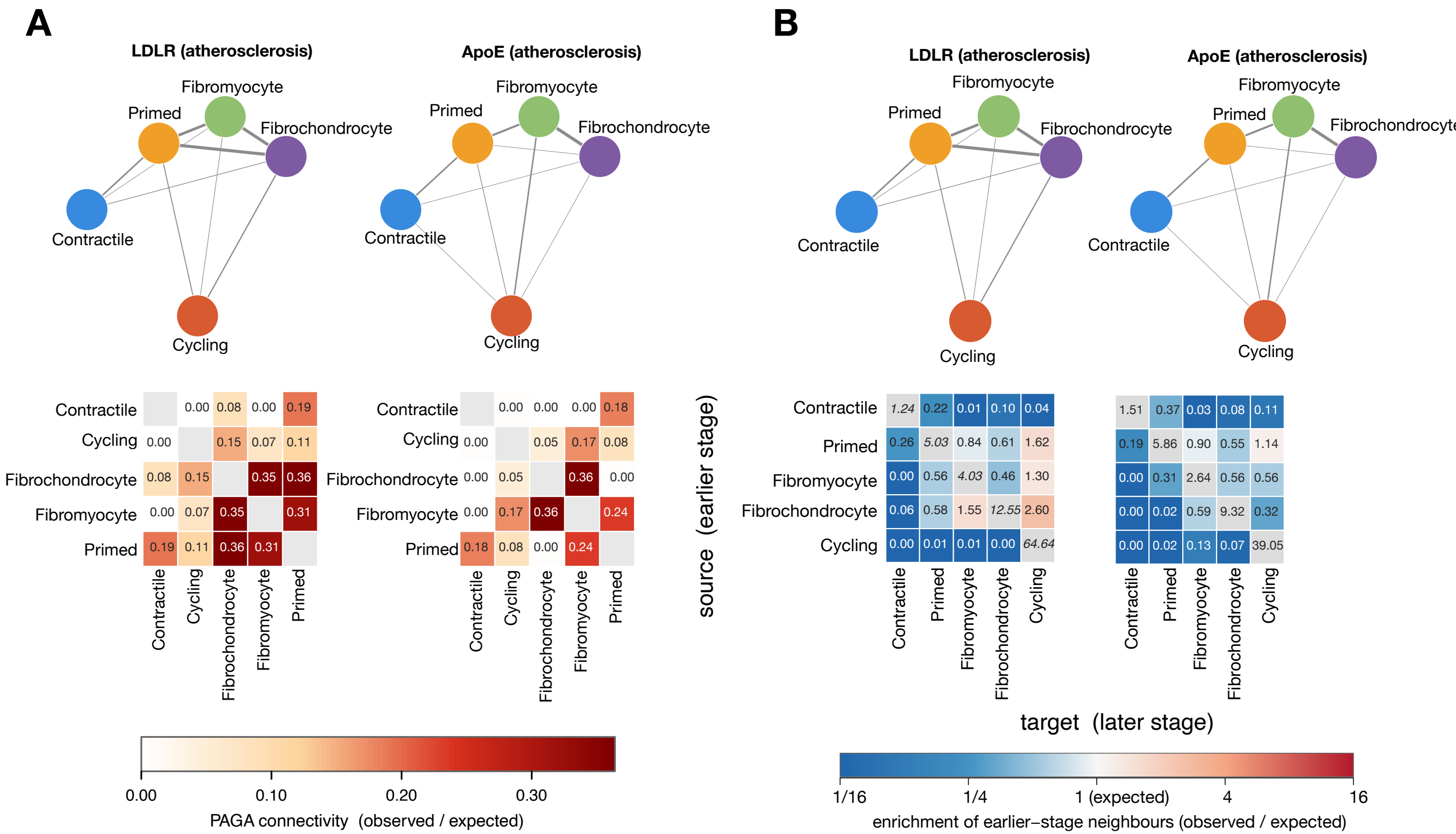
A
LDLR (atherosclerosis)
ApoE (atherosclerosis)
Fibromyocyte
Primed
Fibrochondrocyte
Contractile
Cycling
Contractile 0.00 0.08 0.00 0.19
Cycling 0.00 0.15 0.07 0.11
Fibrochondrocyte 0.08 0.15 0.35 0.36
Fibromyocyte 0.00 0.07 0.35 0.31
Primed 0.19 0.11 0.36 0.31
Contractile 0.00 0.00 0.00 0.18
Cycling 0.00 0.05 0.17 0.08
Fibrochondrocyte 0.00 0.05 0.36 0.00
Fibromyocyte 0.00 0.17 0.36 0.24
Primed 0.18 0.08 0.00 0.24
0.00
0.10
0.20
0.30
PAGA connectivity (observed / expected)
B
source (earlier stage)
Contractile 1.24 0.22 0.01 0.10 0.04
Primed 0.26 5.03 0.84 0.61 1.62
Fibromyocyte 0.00 0.56 4.03 0.46 1.30
Fibrochondrocyte 0.06 0.58 1.55 12.55 2.60
Cycling 0.00 0.01 0.01 0.00 64.64
1.51 0.37 0.03 0.08 0.11
0.19 5.86 0.90 0.55 1.14
0.00 0.31 2.64 0.56 0.56
0.00 0.02 0.59 9.32 0.32
0.00 0.02 0.13 0.07 39.05
target (later stage)
1/16
1/4
1 (expected)
4
16
enrichment of earlier–stage neighbours (observed / expected)
